# *BRRIAR* lncRNA alters breast cancer risk by modulating interferon signaling *in cis* and *in trans*

**DOI:** 10.1186/s12943-025-02510-8

**Published:** 2026-01-07

**Authors:** Haran Sivakumaran, Sneha Nair, Mainá Bitar, Xue Lu, Lu Wang, Ji Liu, Deshapriya S. Karunarathne, P. Prakrithi, Sebastien Jacquelin, Isela Sarahi Rivera, Kristine M. Hillman, Susanne Kaufmann, Rebekah Ziegman, Wei Shi, Sarah Alexandrou, C. Elizabeth Caldon, Rakesh N. Veedu, Quan H. Nguyen, Jonathan Beesley, Michelle N. Wykes, Juliet D. French, Stacey L. Edwards

**Affiliations:** 1 QIMR Berghofer, Brisbane, 4029 Australia; 2 Faculty of Health, Queensland University of Technology, Brisbane, 4006 Australia; 3 School of Biomedical Sciences, The University of Queensland, Brisbane, 4006 Australia; 4 Institute for Molecular Bioscience, The University of Queensland, Brisbane, 4006 Australia; 5 Macrophage Biology Laboratory, Mater Research, Brisbane, 4102 Australia; 6 The Walter and Eliza Hall Institute of Medical Research, Parkville, 3052 Australia; 7 Garvan Institute of Medical Research, Sydney, 2010 Australia; 8 Faculty of Medicine, St Vincent’s Clinical School, UNSW Sydney, Sydney, 2010 Australia; 9 Personalised Medicine Centre, Health Futures Institute, Murdoch University, Perth, 6150 Australia; 10 Precision Nucleic Acid Therapeutics, Perron Institute for Neurological and Translational Science, Perth, 6009 Australia

**Keywords:** Breast cancer, Long noncoding RNA, LncRNA, BRRIAR, BHLHE40, RIG-I, Interferon signaling, Immune response

## Abstract

**Background:**

Interferons (IFNs) are key cytokines that drive immune responses against infections and cancer, yet few therapies have successfully leveraged IFN signaling for cancer treatment. Long noncoding RNAs (lncRNAs) are emerging as promising therapeutic candidates, but their roles in immune modulation remain largely unexplored. Here, we functionally characterize a breast cancer-associated lncRNA, *BRRIAR*, which primes the IFN signaling pathway in specific cancer contexts and represents a potential therapeutic strategy for estrogen receptor-positive (ER+) breast cancer.

**Methods:**

*BRRIAR* expression and subcellular localization were examined using qPCR, in situ hybridization, single-cell RNA sequencing and spatial transcriptomics. *BRRIAR* target genes were identified through CRISPR interference, chromatin interaction assays and ChIP sequencing. Mechanistic studies in ER + breast cancer cells included CRISPR-Cas9 genome-wide screens, RNA sequencing, RNA pull-down followed by mass spectrometry, proliferation assays and Western blotting. The therapeutic potential of *BRRIAR* was evaluated via intratumoral delivery of lipid nanoparticle-encapsulated *BRRIAR* in ER + breast cancer xenograft models. Immune activation was assessed using flow cytometry and cytokine profiling of human peripheral blood mononuclear cells (PBMCs).

**Results:**

We demonstrate that *BRRIAR* is a key target gene at the 3p26 breast cancer risk region. Primarily expressed in ER + breast tumors, *BRRIAR* acts both *in cis* and *in trans.* Nuclear *BRRIAR* regulates *BHLHE40* expression *in cis* through chromatin interactions, while cytoplasmic *BRRIAR* binds *in trans* to the pattern recognition receptor RIG-I, priming IFN signaling. Overexpression of *BRRIAR* RNA triggers RIG-I signaling, inducing IFN responses, drives rapid, dose-dependent apoptosis of ER + breast cancer cells *in vitro* and *in vivo*, and promotes immune activation in human PBMCs.

**Conclusions:**

These findings establish lncRNAs as key regulators of tumor immunity and uncover a critical link between genetic risk, lncRNAs, cancer immunosurveillance and breast cancer development, positioning *BRRIAR* as a promising lncRNA-based RIG-I activator for ER + breast cancer therapy.

**Supplementary Information:**

The online version contains supplementary material available at 10.1186/s12943-025-02510-8.

## Background

Genome-wide association studies (GWAS) have identified 150 genomic regions containing over 5,000 variants associated with breast cancer risk [1, 2]. Most risk variants are located in noncoding regions of the genome, where many modulate distal enhancer function [2, 3]. Another mechanism by which risk variants affect breast cancer risk is through altered expression and/or function of long noncoding RNAs (lncRNAs) [4, 5]. LncRNAs are transcripts longer than 500 nucleotides that do not encode proteins. While the function of most lncRNAs is unknown, characterized lncRNAs often regulate gene expression *in cis* (where the target gene is near the lncRNA locus) or *in trans* (where the target gene is distant) through epigenetic, transcriptional or post-transcriptional mechanisms [6]. We found that breast cancer risk variants are enriched within lncRNA exons and identified 844 risk-associated lncRNAs [4]. CRISPR-Cas13d screening revealed that a subset of these lncRNAs alter breast cancer risk by affecting cell proliferation [7]. While a few have been functionally studied, the biological significance and mechanisms of action for most remain unknown.

Interferons (IFNs) are key cytokines that mediate immune responses against infections and cancer [8, 9]. Type I (α/β) and type III (λ) IFNs are produced when pattern recognition receptors (PRRs) detect pathogen- or damage-associated molecular patterns (PAMPs or DAMPs) [10]. RIG-I (retinoic acid-inducible gene-I; DDX58) is a cytosolic PRR that senses double-stranded RNAs (dsRNAs) with 5’ di- and tri-phosphate ends, a feature common in viral RNAs and some tumor-derived RNAs [11]. RIG-I contains two N-terminal caspase activation and recruitment domains (CARD), a central DExD/H helicase and a C-terminal domain (CTD). When RIG-I binds RNA, it changes shape to expose the CARDs, allowing oligomerization and the formation of phase-separated condensates [12]. These serve as platforms for interacting with MAVS, triggering transcription factors (TFs) and IFN regulatory factors (IRFs) to drive IFN production. Secreted IFNs then activate the JAK/STAT pathway, inducing IFN-stimulated genes (ISGs). RIG-I itself is an ISG, creating a positive feedback loop that amplifies antiviral and antitumor responses. In cancer, this pathways enhances innate immune signaling and promotes recruitment of immune effector cells to eliminate tumor cells [13].

RIG-I can also interact with host-derived lncRNAs under various pathological conditions, resulting in either activation or inhibition of IFN signaling pathways. To date, the best-characterized RIG-I interacting lncRNAs have been identified in mice and are not conserved in humans. For example, *Lnczc3h7a* binds both activated RIG-I and the E3 ligase TRIM25, stabilizing their interaction and promoting K63-linked ubiquitination of RIG-I and IFN signaling [14]. In contrast, *lnc-Lsm3b* interacts with the CTD of RIG-I, competing with viral RNAs and preventing RIG-I activation as part of a negative feedback mechanism to limit IFN production [15]. In humans, several lncRNAs have been shown to regulate RIG-I activity in the context of viral infection. *AVAN*, for example, enhances the interaction between RIG-I and TRIM25, promoting the antiviral response to influenza A virus (IAV) [16]. Additionally, *lncATV* binds to RIG-I and blocks its interaction with viral RNA, suppressing IFN signaling and supporting hepatitis C virus replication [17]. Despite these insights from infection models, the regulation of RIG-I by lncRNAs in human cancers remains largely unexplored.

Here, we characterize a recently identified breast cancer-associated lncRNA, named *BRRIAR* (*BR*east cancer *R*isk *I*TPR1 *A*ntisense *R*NA). We provide evidence that *BRRIAR* regulates BHLHE40 *in cis* and binds to RIG-I *in trans*, modulating ER + breast tumor-intrinsic IFN signaling and promoting an IFN-driven immune response. Overexpression of *BRRIAR* induces dose-dependent apoptosis in ER + breast cancer cells *in vitro* and *in vivo*, while stimulating immune activation in human peripheral blood mononuclear cells (PBMCs) from healthy donors. These findings reveal a critical link between genetic risk, lncRNAs, cancer immunosurveillance and breast tumorigenesis, positioning *BRRIAR* as a promising lncRNA-based RIG-I activator for ER + breast cancer therapy.

## Results

### *BRRIAR* is a target gene at the 3p26 breast cancer risk region

**Fig. 1:**
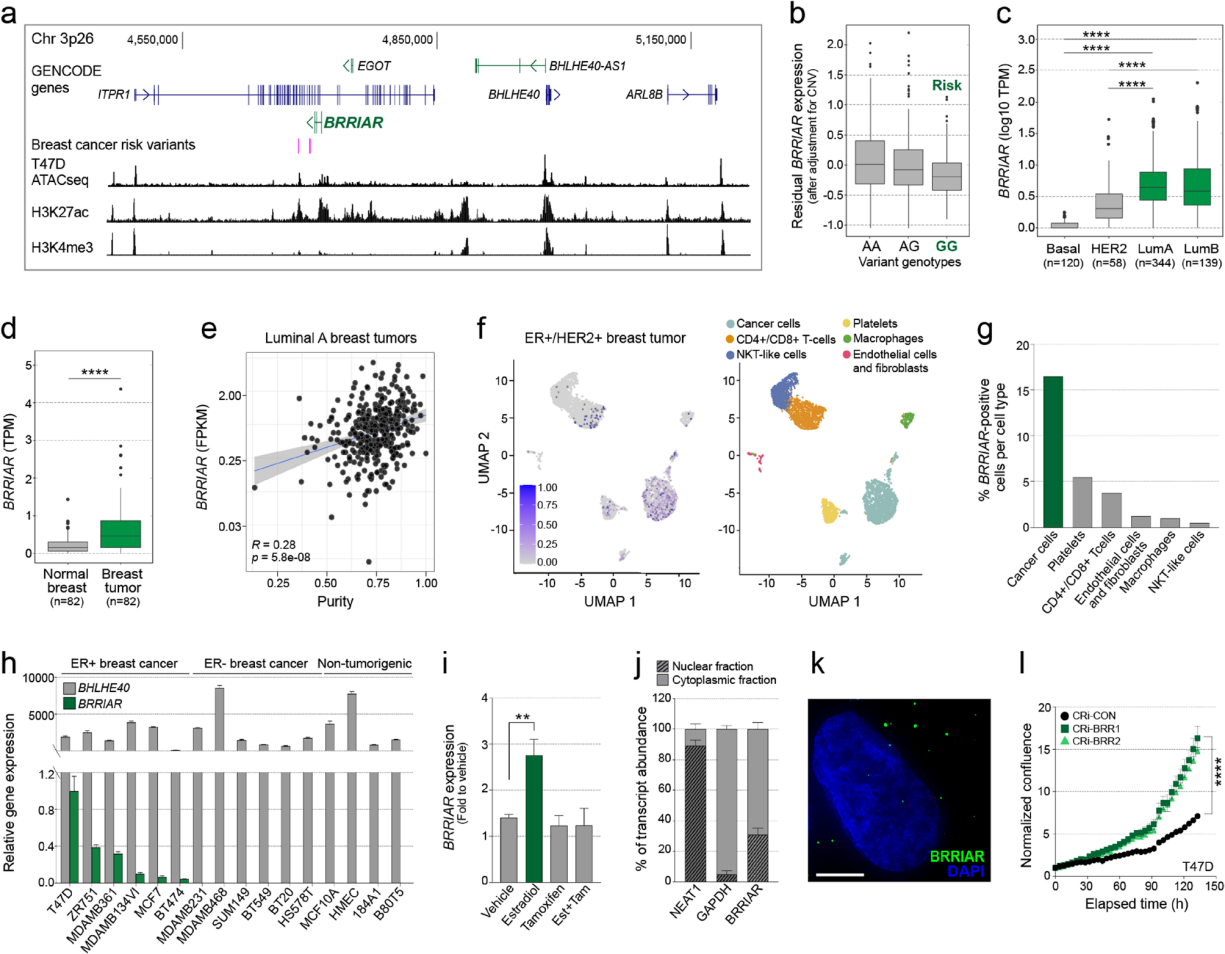
*BRRIAR *is a target gene at the 3p26 breast cancer risk region. **a** WashU genome browser (hg38) showing GENCODE annotated genes (blue) and noncoding RNAs (green). The four GWAS breast cancer risk variants are marked as pink vertical lines. Chromatin accessibility (ATACseq) and histone modification tracks (H3K27ac and H3K4me3) from T47D cells are shown as black histograms. **b** Boxplot of the eQTL analysis and association of breast cancer risk variant rs6762558 with *BRRIAR* expression in TCGA. The *x*-axis of each plot corresponds to the three variant genotypes and the *y*-axis represents log2-normalized gene expression values adjusted for copy number variation (CNV). **c** Boxplot of *BRRIAR* log10 TPM (transcripts per million) in breast tumors from TCGA stratified by tumor subtype. *p* values were determined by Student’s *t-*test (****p < 0.0001). **d** Boxplot of *BRRIAR* TPM in ER + breast tumors and matched adjacent normal breast tissue from TCGA. *p* value was determined by Student’s *t-*test (****p < 0.0001). **e** Scatter plot of *BRRIAR* FPKM (fragments per kilobase million) in luminal A breast tumors from TCGA plotted against estimated tumor purity. The correlation coefficient was calculated using Pearson correlation (*R*), and the *p* value was determined using a two-sided *t*-test. **f** Left panel: UMAP plot of *BRRIAR* expression in scRNAseq data from one representative ER+/HER2 + invasive ductal carcinoma sample. Right panel: cells were categorized into six distinct cell types based on marker gene expression profiles. **g** Percentage of *BRRIAR*-positive cells per cell type. **h** qPCR for *BRRIAR* and *BHLHE40* (nearby gene) expression in ER+, ER- breast cancer and non-tumorigenic breast cell lines. *GAPDH* was used as the internal control. Error bars, SEM (*n* = 3). **i** qPCR for *BRRIAR* expression in T47D cells cultured in charcoal-stripped fetal bovine serum (CS-FBS) for 48 h, followed by treatment with estradiol (10 nM), tamoxifen (1 µM) or estradiol + tamoxifen for 24 h. DMSO was the vehicle control. *GAPDH* was used as the qPCR internal control. Error bars, SEM (*n* = 3). *p* value was determined by one-way ANOVA with Dunnett’s test (**p < 0.01). **j** qPCR after nuclear/cytoplasmic fractionation of T47D cells detecting the distribution of the indicated transcripts. *NEAT1* and *GAPDH* were used as nuclear and cytoplasmic markers, respectively. Error bars, SD (*n* = 2). **k** Representative image of *BRRIAR* in T47D cells stained with Stellaris FISH probes (green). Nuclei were stained with DAPI (blue). Scale bar, 5 μm. **l** Cell confluence in T47D cells measured by IncuCyte after CRISPRi-*BRRIAR*. CRi-CON is a non-targeting control. Error bars, SEM (*n* = 8). *p* value was determined by one-way ANOVA with Dunnett’s test (****p < 0.0001)

GWAS have identified a breast cancer risk region at chromosome 3p26 containing four associated variants (Supplementary Table 1) [2], but the target gene(s) remain unknown. In our previous breast cancer-focused lncRNA study [4], we identified *BRRIAR*, an unannotated, spliced, antisense lncRNA located within introns of *ITPR1* at 3p26 with unknown function (Fig. 1a). Using 5’ and 3’ RACE, we mapped the transcript boundaries and confirmed *BRRIAR* as a 1,106-base pair, three-exon, polyadenylated RNA (Supplementary Table 2). *BRRIAR* shows limited sequence conservation across species but contains two transposable elements, a long terminal repeat (LTR) in exon 2 and a long interspersed element type 1 (LINE1) in exon 3 (Supplementary Fig. 1a). Structural predictions revealed two stem-loop (SL) structures (Supplementary Fig. 1b), a TG-rich SL1 in exon 1 and a GA-rich SL2 in exon 3 which may bind RNA binding proteins based on human CLIP-seq data (Supplementary Fig. 1c) [18, 19]. The *BRRIAR* sequence also contains a putative RNA G-quadraplex (Supplementary Table 2), a motif that facilitates protein binding in other RNAs [20].

Using transcriptomic data from The Cancer Genome Atlas (TCGA), we found that *BRRIAR* acts as an expression quantitative trait locus (eQTL) in breast tumors [4]. The *BRRIAR* eQTL signal colocalized with the GWAS signal, with the risk alleles associated with reduced *BRRIAR* expression (Fig. 1b). *BRRIAR* was more highly expressed in luminal A and luminal B (predominantly ER+) breast tumor subtypes in TCGA (Fig. 1c) [21], and showed a moderate positive correlation with *ESR1* expression in these subtypes (Spearman’s *r* = 0.37, *p* = 2.15e-28). Compared with matched normal breast tissue, *BRRIAR* was overexpressed in ER + breast tumors, although the number of available samples was limited (Fig. 1d). *BRRIAR* expression was positively correlated with luminal A tumor purity, suggesting it is predominantly expressed in epithelial cells (Fig. 1e). No significant correlations were observed in luminal B, HER2 or basal subtypes (Supplementary Fig. 1d). Single-cell RNA sequencing (scRNAseq) of an ER+/HER2 + invasive ductal carcinoma sample further confirmed that *BRRIAR* expression was largely restricted to tumor cells (16.5%), with only sparse expression detected in platelets (5.5%), CD4+/CD8 + T-cells (3.8%), and other cell types (Fig. 1f, g and Supplementary Fig. 1e-g and Supplementary Tables 3–5). Of note, although limited in number, immune cells displayed higher *BRRIAR* expression than most tumor cells at the single-cell level (Supplementary Fig. 1h).

Consistent with these findings, *BRRIAR* was primarily expressed in ER + breast cancer cell lines (Fig. 1h and Supplementary Fig. 1i) and was upregulated at later time points following estradiol stimulation in T47D cells (Supplementary Fig. 1j). This induction was diminished by tamoxifen or combined estrogen and tamoxifen treatment, indicating that *BRRIAR* is ESR1-dependent but likely regulated through an indirect mechanism (Fig. 1i and Supplementary Fig. 1k). Given that *BRRIAR* expression is highest in T47D cells, we examined its subcellular localization in this line and found that it is present in both the nucleus and cytoplasm (Fig. 1j, k). Functionally, *BRRIAR* emerged as a positive hit in our recent CRISPR-Cas13d screens [7], where its knockdown increased the proliferation of ER + breast cancer cells. To validate this result, we independently silenced *BRRIAR* using CRISPR-interference (CRISPRi) with dCas9-KRAB and two guide RNAs (gRNAs) targeting its promoter (Supplementary Fig. 1l). Consistent with our screens, CRISPRi-mediated *BRRIAR* silencing significantly increased proliferation of T47D and MCF7 cells compared to CRISPRi-control cells (Fig. 1l and Supplementary Fig. 1m). Collectively, these findings identify *BRRIAR* as a key functional target gene at the 3p26 breast cancer risk region.

### *BRRIAR* is an elncRNA that affects the activity of an enhancer cluster

**Fig. 2:**
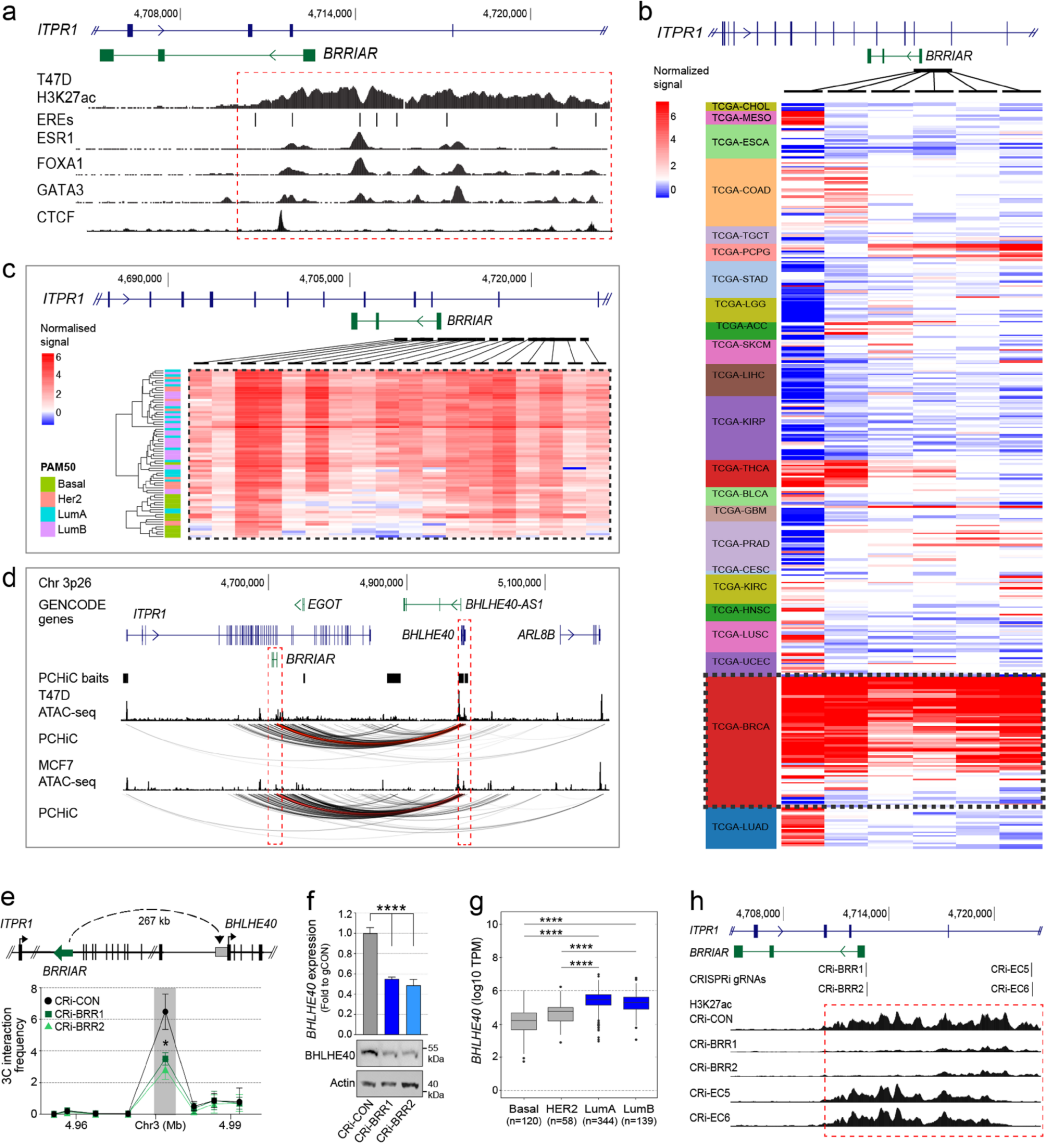
*BRRIAR* acts *in cis* to modulate the enhancer cluster and BHLHE40.** a** WashU genome browser (hg38) showing *ITPR1* (blue) and *BRRIAR* (green). H3K27ac, ESR1, FOXA1, GATA3 and CTCF tracks for T47D cells are shown as black histograms. JASPAR CORE 2024-predicted estrogen response elements (EREs) are shown as black vertical lines. The dashed red outline marks the ~ 11 kb enhancer cluster. **b** Heatmap of ATACseq predicted peak-to-gene links across 23 cancer types, expressed as z-scores. Each column represents ATACseq predicted peaks shared across multiple cancer types within the *BRRIAR*-associated enhancer cluster. **c** Zoomed in heatmap of ATACseq predicted peak-to-gene links specific to breast tumors (TCGA-BRCA) stratified by the PAM50 subtypes (n = 74 donors), expressed as z-scores. Each column represents the subset of ATACseq predicted peaks present in all breast tumor samples. **d** WashU genome browser (hg38) showing annotated genes (blue) and noncoding RNAs (green). The promoter capture HiC (PCHiC) baits are marked as black boxes. The ATAC-seq tracks are shown as black histograms. PCHiC interactions are shown as arcs. The dashed red outlines and red arcs highlight chromatin looping between *BRRIAR* and the *BHLHE40* promoter. **e** 3 C interaction profiles between *BRRIAR* and the *BHLHE40* promoter in T47Ds after CRISPRi-*BRRIAR*. Error bars, SEM (n = 3). *p* value was determined by one-way ANOVA with Tukey test (*p < 0.05). **f** qPCR and Western blot for *BHLHE40* expression in T47D cells after CRISPRi-*BRRIAR*. *EIF2B1* was used as the qPCR internal control. Error bars, SEM (n = 3). *p* values were determined by one-way ANOVA with Dunnett’s test (****p < 0.0001). Actin was used as the Western loading control. **g** Boxplot of *BHLHE40* log10 TPM in breast tumors from TCGA stratified by tumor subtype. *p* values were determined by Student’s *t-*test (****p < 0.0001). **h** WashU genome browser (hg38) showing *ITPR1* (blue) and *BRRIAR* (green). H3K27ac ChIPseq data in T47D cells after CRISPRi-*BRRIAR* or using gRNAs targeted to the 3’ end of the enhancer cluster (CRi-EC5/6) are shown as black histograms. The dashed red outline marks the ~ 11 kb enhancer cluster

LncRNAs transcribed from enhancers (known as elncRNAs) often play critical roles in gene regulation [22]. When we analyzed histone modification data from ER + breast cancer cells, we found that the transcription start site of *BRRIAR* lies within an ~ 11 kb enhancer cluster exhibiting high H3K27ac signal in *BRRIAR*-expressing T47D cells (Fig. 2a). The enhancer cluster contains multiple binding sites for TFs involved in estrogen signaling including ESR1, FOXA1 and GATA3, as well as CTCF motifs consistent with enhancer looping. This enhancer cluster is also present in other ER + breast cancer cells that express *BRRIAR*, where H3K27ac levels align with *BRRIAR* expression. In contrast, breast cell lines with no *BRRIAR* expression lack H3K27ac marks in the region (Supplementary Fig. 2a). Genome-wide chromatin accessibility profiles (ATACseq) from 410 tumor samples across 23 cancer types [23] showed that the enhancer cluster has ATACseq peaks largely restricted to breast tumors (Fig. 2b). When breast tumors were stratified by their PAM50 intrinsic molecular signatures, these ATACseq peaks were most prominent in luminal A and luminal B subtypes (Fig. 2c).

### The* BRRIAR* locus/enhancer cluster regulates BHLHE40 expression *in cis*

Given that lncRNAs often regulate nearby genes *in cis*, we examined promoter CHiC data previously generated in our lab from breast cells to determine whether the *BRRIAR* locus or its associated enhancer cluster physically interacts with local promoters [3]. We observed strong chromatin interactions between *BRRIAR* and the *BHLHE40* promoter, located ~ 267 kb away, in T47D and MCF7 cells, with no interactions detected with other baited gene promoters in these cells (Fig. 2d). To determine whether *BRRIAR* modulates this chromatin looping, we performed 3 C-qPCR in T47D cells after CRISPRi-*BRRIAR* silencing. This significantly reduced chromatin looping between the *BRRIAR* locus and *BHLHE40* promoter (Fig. 2e and Supplementary Fig. 2b), accompanied by decreased BHLHE40 expression, suggesting *BRRIAR* regulates *BHLHE40* in ER + breast cancer cells (Fig. 2f and Supplementary Fig. 2c). Using the same 3 C libraries, we found no change in chromatin looping between a nearby, unrelated H3K27ac-marked enhancer (~ 25 kb from *BRRIAR*) and either the *BRRIAR* or *BHLHE40* promoters (Supplementary Fig. 2d), supporting the specificity of the *BRRIAR-BHLHE40* regulatory interaction. *BHLHE40* (basic helix-loop-helix family member e40) was also highly expressed in luminal A and luminal B breast tumor subtypes in TCGA (Fig. 2g), and showed a mild positive correlation with *BRRIAR* expression in these subtypes (Spearman’s r = 0.25, *p* = 7.08e-13). Similar to ER + breast cancer cells, ER-negative and non-tumorigenic breast cell lines, which lack *BRRIAR* expression but retain H3K27ac binding at the 5’ and 3’ ends of the enhancer cluster (Supplementary Fig. 2a), showed strong interactions between the enhancer and *BHLHE40*, and a weaker interaction with *EDEM1* (Supplementary Fig. 2e). *ARL8B* may also be a target, but its promoter region could not be baited. While chromatin looping alone does not establish function, these findings suggest the enhancer cluster may regulate additional genes *in cis*.

To investigate whether *BRRIAR* modulates the epigenetic state of the enhancer cluster, we performed H3K27ac chromatin immunoprecipitation sequencing (ChIPseq) in T47D cells after CRISPRi-mediated repression of the *BRRIAR* promoter. This resulted in a significant reduction of H3K27ac levels across the entire ~ 11 kb enhancer cluster compared to the CRISPRi-control cells (Fig. 2h). To determine whether this effect was a general consequence of CRISPRi targeting, we also performed H3K27ac ChIPseq or ChIP-qPCR in T47D cells using gRNAs directed to two individual H3K27ac peaks within the enhancer cluster and to its 3’ end. Across all additional gRNAs, CRISPRi led to localized reduction of H3K27ac at the targeted sites without affecting *BRRIAR* expression (Fig. 2h and Supplementary Fig. 2f, g). In agreement with the chromatin looping data, CRISPRi suppression of one targeted H3K27ac peak within the enhancer cluster also reduced *BHLHE40* expression (Supplementary Fig. 2f, g). Together, these findings suggest that *BRRIAR* transcription and/or *BRRIAR* RNA is required to maintain H3K27ac across the full ~ 11 kb enhancer cluster. Genome-wide analysis of the H3K27ac ChIPseq data identified 122 differentially acetylated regions (DARs; FDR < 5%) between the CRISPRi-*BRRIAR* and CRISPRi-control cells, including 49 (40%) mapping to protein-coding gene promoters (Supplementary Table 6). Gene ontology analysis indicated that the DARs were enriched for IFN signaling-related genes (Supplementary Fig. 2h and Supplementary Table 7). CRISPRi-*BRRIAR* induced coordinated changes in H3K27ac and gene expression, with a strong positive correlation between enhancer activity and transcriptional output (Spearman’s *r* = 0.88, *p* = 2.3e-11; Supplementary Fig. 2i). DARs were also enriched (FDR < 5%) for class B E-boxes, the DNA motif recognized by BHLHE40, and for PU.1 binding sites, which are regulated by IFN/immune TFs IRF4 and IRF8 [24] (Supplementary Table 8).

### The *BRRIAR* RNA regulates *BHLHE40 *expression* in cis*

At some lncRNA loci, the transcribed RNA product is functional rather than the act of transcription. To characterize *BRRIAR* as a functional RNA, we used 2’-*O*-methoxyethyl antisense oligonucleotides (ASOs) to target and cleave the *BRRIAR* RNA without affecting its DNA element. Two independent ASOs were designed to accessible regions identified by RNase H-based cleavage of RNA-DNA-heteroduplexes (Supplementary Fig. 3a). To determine whether *BRRIAR* RNA knockdown modulates the enhancer cluster, we performed H3K27ac ChIP-qPCR in T47D cells transfected with *BRRIAR*-targeting ASOs. We observed no significant change in H3K27ac signal across the enhancer cluster compared to ASO-control cells (Supplementary Fig. 3b, c), suggesting that the H3K27ac signal is maintained by the act of *BRRIAR* transcription. In contrast, *BRRIAR* knockdown with ASOs reduced chromatin looping between the *BRRIAR* locus and the *BHLHE40* promoter, resulting in decreased *BHLHE40* expression (Supplementary Fig. 3d, e). These findings indicate that *BRRIAR* RNA facilitates chromatin looping to regulate *BHLHE40* expression.

### *BRRIAR* modulates IFN signaling in ER + breast cancer cells

**Fig. 3:**
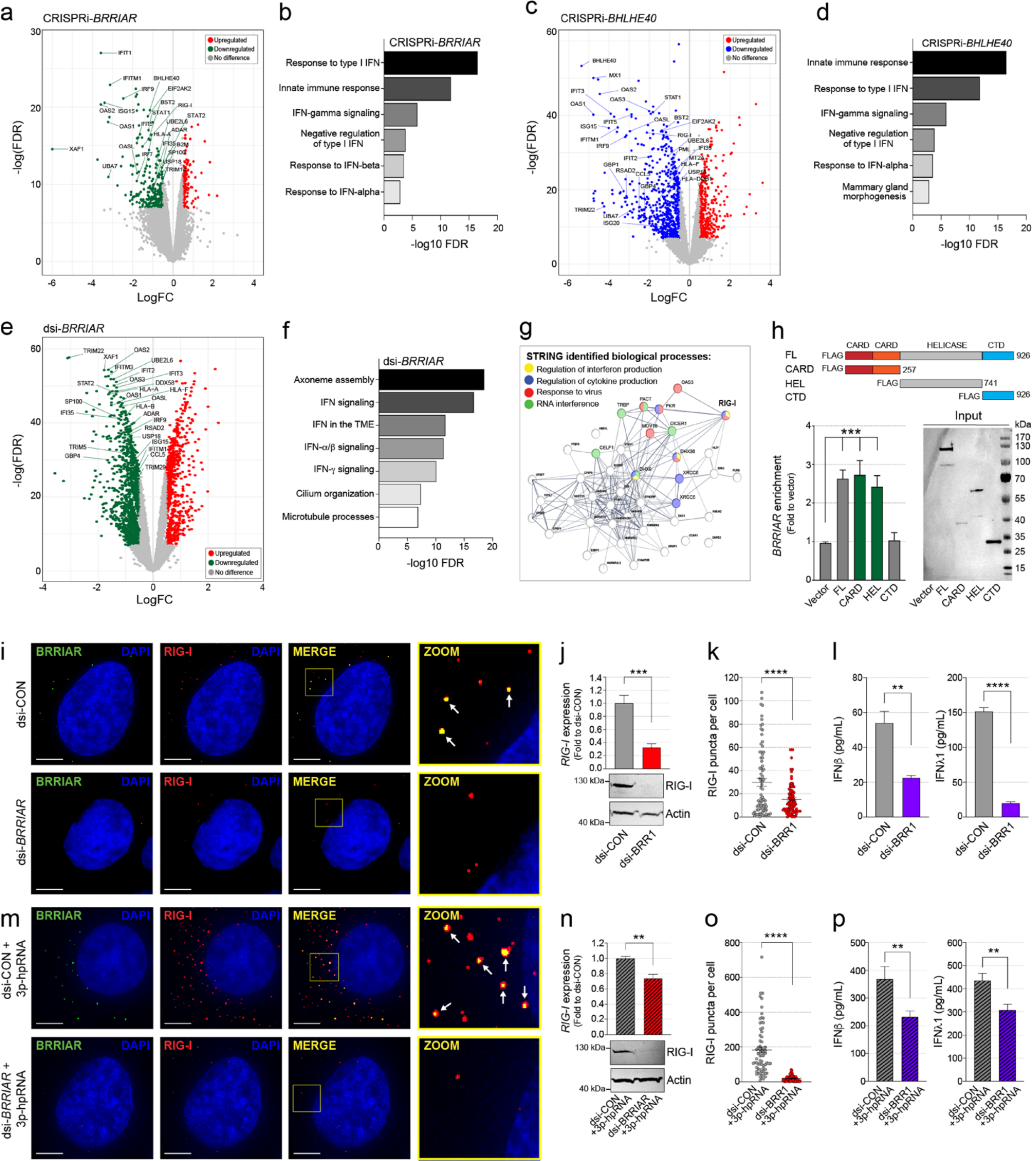
*BRRIAR* modulates IFN signaling pathways in ER + breast cancer cells.** a**,** c** Volcano plots showing differentially expressed genes (DEGs) in T47D cells after CRISPRi-*BRRIAR* (CRi-*BRR1*) or CRISPRi-*BHLHE40* (CRi-*BHL1*). Significant DEGs are shown as green, red or blue dots. **b**,** d** Pathway enrichment of DEGs. **e** Volcano plot showing DEGs in T47D cells after dsi-CON or dsi-*BRRIAR* (dsi-*BRR1*) treatment for 48 h. Significant DEGs are shown as green and red dots. **f** Pathway enrichment of DEGs. **g** STRING network interaction image for the top enriched *BRRIAR*-binding proteins. **h** RIP for binding of *BRRIAR* to FLAG-RIG-I or FLAG-tagged truncation mutants of RIG-I from T47D cells. Top: Schematic of FLAG-RIG-I truncation mutants: FL, full-length RIG-I; CARD, N-terminal caspase activation and recruitment domain; HEL, DExD/H helicase domain; CTD, C-terminal domain. Left: Copurified RNA from FLAG-RIG-I IPs assayed for *BRRIAR* enrichment by qPCR. *GAPDH* was used as the internal control. Error bars, SEM (*n* = 3). *p* values were determined by one-way ANOVA with Dunnett’s test (***p < 0.001). Right: IP specificity was controlled by FLAG Western blotting. **i** Representative microscopy images of *BRRIAR* (green) and RIG-I (red) after dsi-CON or dsi-*BRR1* treatment for 48 h. The dsi-CON is a non-targeting control. Nuclei were stained with DAPI (blue). Scale bars, 5 μm. Zoom: white arrows highlight *BRRIAR*/RIG-I co-localization. **j** qPCR and Western blot for RIG-I in T47D cells after dsi-CON or dsi-*BRR1* treatment for 48 h. *EIF2B1* was used as the qPCR internal control. Error bars, SEM (*n* = 3). *p* value was determined by Student’s *t-*test (***p < 0.001). Actin was used as the Western loading control. **k** Plot of the RIG-I puncta per cell (*n* = 100). Error bars, SEM (*n* = 3). *p* values were determined by Student’s *t-*test (****p < 0.0001). **l** ELISA for IFNβ and IFNλ1 secreted from T47D cells after dsi-CON or dsi-*BRRIAR* treatment. Error bars, SEM (*n* = 3). *p* value was determined by Student’s *t-*test (**p < 0.01, ****p < 0.0001). **m** Representative microscopy images of *BRRIAR* (green) and RIG-I (red) in T47D cells after dsi-CON or dsi-*BRR1* treatment for 48 h followed by exposure to 3p-hpRNA (0.5 µg/ml) for 6 h. Nuclei were stained with DAPI (blue). Scale bars, 5 μm. Zoom: white arrows highlight *BRRIAR*/RIG-I co-localization. **n** qPCR and Western blot for RIG-I in T47D cells after dsi-CON or dsi-*BRR1* treatment for 48 h followed by exposure to 3p-hpRNA (0.5 µg/ml) for 6 h. *EIF2B1* was used as the qPCR internal control. Error bars, SEM (*n* = 3). *p* value was determined by Student’s *t-*test (**p < 0.01). Actin was used as the Western loading control. **o** Plot of the RIG-I puncta per cell after exposure to 3p-hpRNA (0.5 µg/ml) for 6 h (*n* = 100). Error bars, SEM (*n* = 3). *p* value was determined by Student’s *t-*test (****p < 0.0001). **p** ELISA for IFNβ and IFNλ1 secreted from T47D cells after dsi-CON or dsi-*BRR1* treatment for 48 h followed by exposure to 3p-hpRNA (0.5 µg/ml) for 6 h. Error bars, SEM (*n* = 3). *p* values were determined by Student’s *t-*test (**p < 0.01)

To gain insight into the functions of *BRRIAR* and *BHLHE40* in ER + breast cancer cells, we next performed RNAseq on T47D cells after CRISPRi-*BRRIAR* or CRISPRi-*BHLHE40* repression (Supplementary Fig. 3f). We identified 310 (|logFC| >0.5 and FDR < 1%) and 383 (|logFC| >1.0 and FDR < 1%) differentially expressed genes (DEGs) between the CRISPRi-*BRRIAR* or CRISPRi-*BHLHE40* and CRISPRi-control cells, respectively (Fig. 3a, c and Supplementary Fig. 3g and Supplementary Table 9). Of these, *BHLHE40* was the only gene *in cis* (within 1 Mb) whose expression was significantly altered by CRISPRi-*BRRIAR*. Forty-seven DEGs overlapped between CRISPRi-*BRRIAR* and CRISPRi-*BHLHE40* (Fisher’s exact test; *p-value* < 6.4e-50), suggesting that the two genes are within shared regulatory pathways. Pathway analysis of downregulated DEGs in both CRISPRi-*BRRIAR* and CRISPRi-*BHLHE40* cells showed significant enrichment for IFN signaling and innate immune response pathways (FDR < 1%; Fig. 3b, d and Supplementary Fig. 3h). In contrast, upregulated DEGs in CRISPRi-*BRRIAR* cells were enriched in cell cycle-related processes (FDR < 1%; Supplementary Fig. 3h and Supplementary Table 10), potentially explaining the increased proliferation of T47D and MCF7 cells following *BRRIAR* depletion (Fig. 1l).

### Cytoplasmic *BRRIAR* modulates IFN signaling

*BRRIAR* depletion using either CRISPRi or ASOs also suppressed *BHLHE40* expression. Given that *BRRIAR* RNA is predominantly cytoplasmic, we used Dicer-substrate small interfering RNAs (dsiRNAs) to investigate its cytoplasmic function. Silencing *BRRIAR* with two independent dsiRNAs markedly reduced mature *BRRIAR* RNA levels in the cytoplasm of T47D and ZR751 cells (Supplementary Fig. 4a, b). In contrast, because mature *BRRIAR* RNA was not reliably detected in nuclear fractions, heterogeneous nuclear (*hn*) *BRRIAR* RNA was quantified and showed no significant change compared to the dsi-control (Supplementary Fig. 4c). Similarly, *BHLHE40* levels remained unaffected (Supplementary Fig. 4d), confirming that both dsiRNAs selectively target and cleave cytoplasmic *BRRIAR* RNA. RNAseq on T47D cells after dsi-*BRRIAR* identified 437 DEGs (|logFC| >0.5 and p-value < 0.001) relative to dsi-control cells (Fig. 3e and Supplementary Fig. 4e and Supplementary Table 11). While upregulated DEGs showed no pathway enrichment, downregulated DEGs were again strongly enriched for IFN signaling pathways (FDR < 1%; Fig. 3f and Supplementary Fig. 4f and Supplementary Table 12). qPCR validation in ZR751 cells confirmed downregulation of several ISGs following dsi-*BRRIAR* silencing (Supplementary Fig. 4g), indicating that this effect is not restricted to T47D cells. These findings suggest that cytoplasmic *BRRIAR* modulates IFN signaling in ER + breast cancer cells independently of *BHLHE40*.

### Cytoplasmic *BRRIAR* regulates RIG-I activation

Growing evidence suggests that endogenous lncRNAs can bind cytosolic proteins to promote or inhibit downstream IFN signaling. To identify *BRRIAR*-associated proteins, we performed an RNA pull-down assay using *in vitro* transcribed (IVT) *BRRIAR* and T47D cell lysates, followed by mass spectrometry. Proteins were ranked based on enrichment relative to an IVT control (*LacZ*; Supplementary Table 13). Among the top *BRRIAR*-associated proteins (p-value < 0.05), 12 are involved in the regulation of IFN or cytokine production (Fig. 3g). The strongest (top statistically enriched) interactor was RIG-I, which also showed marked functional correlation with the other IFN-related proteins (FDR < 1%). To validate and map this interaction, we performed RNA immunoprecipitation (RIP) in T47D cells, which showed that *BRRIAR* preferentially associated with the CARD and helicase domains of RIG-I (Fig. 3h), distinct from the CTD responsible for viral RNA binding [11]. Combined RNA-fluorescence in situ hybridization (FISH) and immunofluorescence (IF) revealed distinct cytoplasmic RIG-I puncta in dsi-control T47D cells, which likely represent phase-separated condensates [12], most of which co-localized with *BRRIAR* (Fig. 3i and Supplementary Fig. 4h, i). Silencing *BRRIAR* with dsiRNAs led to the reduction of RIG-I levels, the number of puncta per cell, and type I/III IFN levels (Fig. 3i-l), suggesting that *BRRIAR* may contribute to mild RIG-I activation in these cells. To determine whether *BRRIAR* also modulates RIG-I activation, we treated T47D cells with 3p-hpRNA, a potent synthetic RIG-I agonist [25]. As expected, 3p-hpRNA treatment significantly increased RIG-I levels, puncta formation and IFN production in dsi-control T47D cells, but these responses were diminished in *BRRIAR*-depleted cells (Fig. 3m-p and Supplementary Fig. 4j, k). Similar results were observed with 5’ppp-dsRNA, another synthetic RIG-I agonist (Supplementary Fig. 4l-n). As the dsiRNAs primarily silence cytoplasmic *BRRIAR*, the observed effects are unlikely to result from direct transcriptional regulation of RIG-I, but rather impaired RIG-I activation and downstream IFN signaling.

### *BRRIAR* overexpression triggers ER + breast cancer cell death *in vitro*

**Fig. 4:**
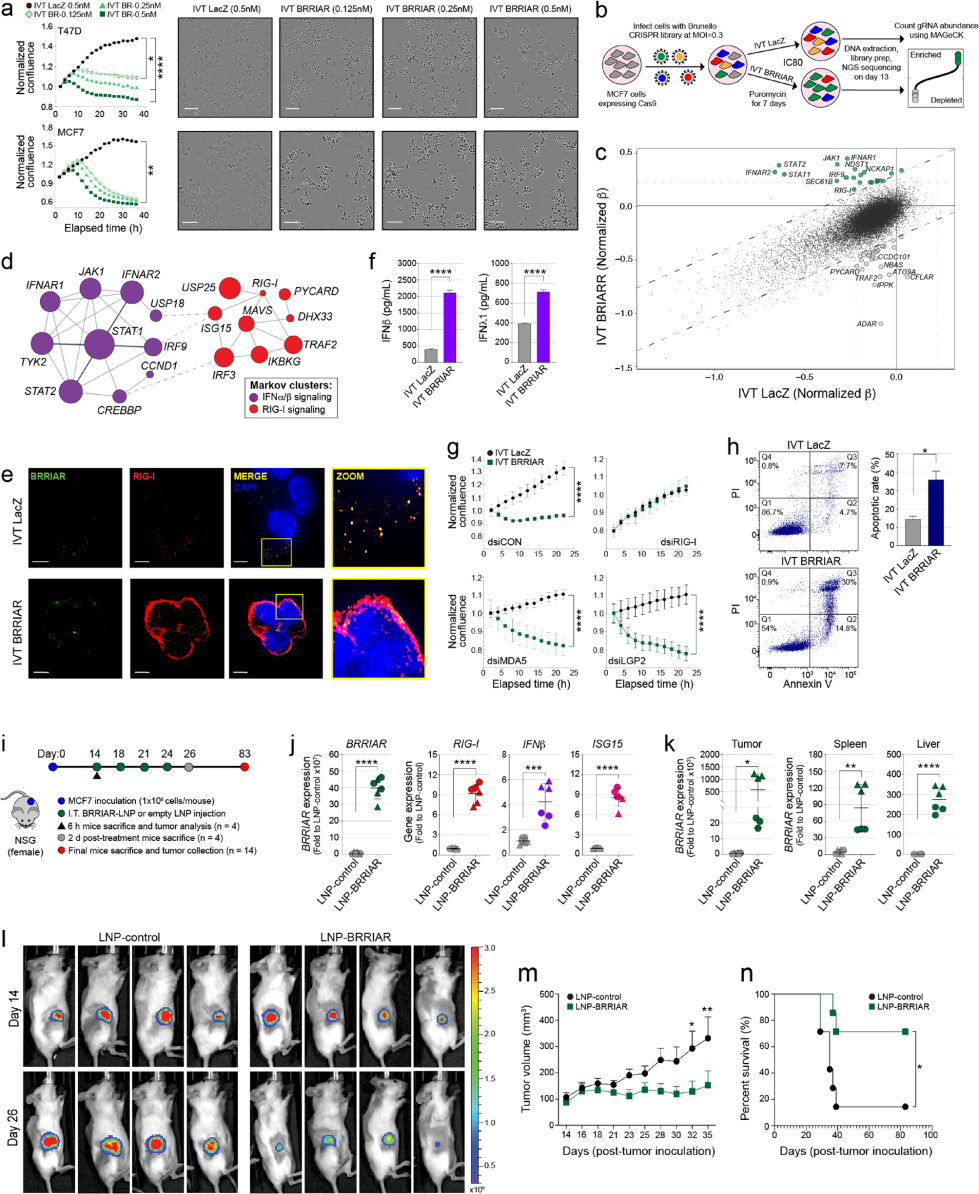
*BRRIAR* induces RIG-I-mediated apoptosis in ER + breast cancer cells.** a** Left panels: cell confluence in T47D and MCF7 cells measured by IncuCyte after transfection of IVT *LacZ* (0.5 nM) or IVT *BRRIAR* (0.125 nM, 0.25 nM, 0.5 nM). Error bars, SEM (*n* = 4). *p* values were determined by one-way ANOVA with Dunnett’s test (*p < 0.05, **p < 0.01, ****p < 0.0001). Right panels: representative IncuCyte images. Scale bar, 100 μm. **b** Schematic workflow of genome-wide CRISPR-Cas9 screens in MCF7 cells. **c** Gene-level effects (normalized β values); enriched (green circles) or depleted (gray circles) genes for response to IVT *BRRIAR* versus IVT *LacZ* exposure are labelled. The dotted lines indicate ± 2 standard deviations around the fitted linear regression line. **d** STRING network analysis of genes that were enriched or depleted with a FDR < 0.2 in IVT *BRRIAR* versus IVT *LacZ*. Lines indicate confidence and circle size is scaled by β effects detected in the screen. Selected clusters were determined by Markov clustering. **e** Representative microscopy images of *BRRIAR* (green) and RIG-I (red) in T47D cells after transfection of IVT *LacZ* or IVT *BRRIAR* (0.5 nM) for 3 h. Nuclei were stained with DAPI (blue). Scale bars, 5 μm. Zoom: white arrows highlight *BRRIAR*/RIG-I co-localization. **f** ELISA for IFNβ and IFNλ1 secreted from T47D cells after transfection of IVT *LacZ* or IVT *BRRIAR* (0.5 nM) for 3 h. Error bars, SEM (*n* = 3). *p* values were determined by Student’s *t-*test (****p < 0.0001). **g** Cell confluence in T47D cells measured by IncuCyte after co-transfection with indicated dsi-RNAs and IVT *LacZ* or IVT *BRRIAR* (0.5 nM). Error bars, SEM (*n* = 4). *p* values were determined by one-way ANOVA with Dunnett’s test (****p < 0.0001). **h** Left panels: apoptosis analysis of T47D cells after transfection of IVT *LacZ* or IVT *BRRIAR* (0.2 nM) for 24 h by double staining with Annexin V (AV) and propidium iodide (PI). The quadrants (Q) were defined as Q1 = live (AV-/PI-negative), Q2 = early stage of apoptosis (AV-positive/PI-negative), Q3 = late stage of apoptosis (AV-/PI-positive) and Q4 = necrosis (AV-negative/PI-positive). Right panel: percentage of cells in early and late-stage apoptosis in each group (Q2 + Q3). Error bars, SEM (*n* = 3). *p* value was determined by Student’s *t-*test (*p < 0.05). **i** LNP-*BRRIAR* treatment schedule for *in vivo* experiments. Four doses of LNP-*BRRIAR* or LNP-control (empty LNPs; 0.5 mg/kg) were injected intratumorally (I.T.) per mouse. **j** qPCR for *BRRIAR* and ISG expression in MCF7 tumors from mice 6 h after a single I.T. injection of LNP-*BRRIAR* or LNP-control. *GAPDH* was used as the internal control. Error bars, SD. The shapes represent data from different mice. *p* values were determined by Student’s *t-*test (***p < 0.001, ****p < 0.0001). **k** qPCR for *BRRIAR* expression in MCF7 tumors, spleen and liver from mice, 2 days after the last I.T. injection of LNP-*BRRIAR* or LNP-control. *GAPDH* was used as the internal control. Error bars, SD. The shapes represent data from different mice. *p* values were determined by Student’s *t-*test (**p < 0.01, ****p < 0.0001). **l** Bioluminescence intensities of MCF7 tumors in mice imaged before (day 14) and after (day 26) I.T. injections of LNP-*BRRIAR* or LNP-control. **m** MCF7 tumor growth curves in mice treated with LNP-*BRRIAR* or LNP-control. Error bars, SEM (*n* = 7 mice per group). *p* values were determined by two-way ANOVA with Sidak’s test (*p < 0.05, **p < 0.01). **n** Kaplan-Meier survival curves for mice treated with LNP-*BRRIAR* or LNP-control (*n* = 7 mice per group). *p* value was determined by log-rank Mantel-Cox test (*p < 0.05)

Endogenous *BRRIAR* is expressed at low levels across all analyzed ER + breast cancer cell lines. To further explore its function, we introduced *BRRIAR* into the cytoplasm by either directly adding IVT *BRRIAR* to the culture medium or by transfection. Direct addition of IVT *BRRIAR* or control IVT RNA (*LacZ*) had no effect on cell growth or IFNβ production (Supplementary Fig. 5a, b), suggesting that Toll-like receptor 3 (TLR3), a transmembrane receptor that detects extracellular dsRNA to trigger immune responses, was not activated [26]. In contrast, transfection of IVT *BRRIAR* triggered rapid cell death in T47D, MCF7 and ZR751 cells at all tested doses compared to the IVT *LacZ* (Fig. 4a and Supplementary Fig. 5c, d). This effect was independent of ER, as T47D cells depleted of estrogen by 48 h of culture in medium containing CS-FBS were equally sensitive to IVT *BRRIAR* (Supplementary Fig. 5e). To benchmark the potency of IVT *BRRIAR*, we directly compared it to 3p-hpRNA. IVT *BRRIAR* was over 50-fold more potent than 3p-hpRNA in inducing cell death in both T47D and MCF7 cells (Supplementary Fig. 5f). Given the clinical significance of tamoxifen and combination therapies, along with the emergence of resistance in ER + breast cancer [27], we also assessed the effect of IVT *BRRIAR* in drug-resistant models. Transfection of IVT *BRRIAR* into tamoxifen-resistant T47D and MCF7 cells, as well as MCF7 cells resistant to both tamoxifen and the CDK4/6 inhibitor palbociclib, induced levels of cell death comparable to parental lines, indicating that *BRRIAR* remains effective even in the context of therapy resistance. (Supplementary Fig. 5c, g,h). IVT RNA retains a 5’ppp moiety, a key structural feature of viral RNAs that activates RIG-I [28]. To test whether the 5’ structure is required for RIG-I activation by *BRRIAR*, IVT *BRRIAR* transcripts were capped with an anti-reverse cap analog (ARCA) before transfection into T47D cells. Capped IVT *BRRIAR* induced T47D cell death as effectively as unmodified IVT *BRRIAR* (Supplementary Fig. 5i). These results demonstrate that transfection of IVT *BRRIAR* induces robust cell death in ER + breast cancer cell models, regardless of its 5’ end modification.

To explore the mechanisms by which IVT *BRRIAR* induces cell death, we conducted whole-genome CRISPR-Cas9 knockout screens in MCF7 cells treated with IVT *BRRIAR* (Fig. 4b and Supplementary Fig. 6a, b). The gRNAs for numerous IFN-related genes were significantly enriched in IVT *BRRIAR*-treated cells as compared to IVT *LacZ*-treated control cells, including *IFNAR1/2*,

*JAK1/TYK2* and *STAT1/2*. The most significant negative hits were *ADAR*,* IPPK* and *CFLAR* (Fig. 4c and Supplementary Table 14). Markov clustering of STRING network annotations for significantly enriched or depleted genes (FDR < 0.2) highlighted key roles for IFNα/β and RIG-I signaling (Fig. 4d and Supplementary Table 15). To validate these observations, we performed RNA-FISH and IF in T47D cells treated with IVT *LacZ* or IVT *BRRIAR*. While IVT *LacZ* mildly activated RIG-I and IFN production, IVT *BRRIAR* induced a robust upregulation of RIG-I expression (Fig. 4e) and type I/III IFN production (Fig. 4f and Supplementary Fig. 6c). Additionally, dsiRNA-mediated silencing of RIG-I blocked IVT *BRRIAR*-induced cell death, whereas silencing of two other PRRs, MDA5 and LGP2, had no effect (Fig. 4g and Supplementary Fig. 6d). Consistent with RIG-I-mediated apoptosis, T47D cell death showed increased annexin V staining and activation of effector caspases (Fig. 4h and Supplementary Fig. 6e).

### *BRRIAR *overexpression triggers ER + breast cancer cell death *in vivo *

To evaluate the potential of IVT *BRRIAR* to induce apoptosis *in vivo*, we encapsulated 5’capped (cap-1) IVT *BRRIAR* into SM-102-based lipid nanoparticles (LNP-*BRRIAR*) [29]. Delivery efficiency was initially tested *in vitro* by treating MCF7 cells with LNP-*BRRIAR*, which led to significant cell death, increased ISG expression and IFNβ production compared to the empty LNP-control (Supplementary Fig. 6f-i). For the *in vivo* studies, MCF7-luciferase-expressing cells were injected into the mammary fat pads of female NSG mice pre-implanted with estrogen pellets. Once tumors reached ~ 50 mm^3^ in size, mice received four intratumoral injections of either LNP-control or LNP-*BRRIAR* at a dose of 10 µg per mouse (~ 0.5 mg/kg; Fig. 4i). Six hours after the first dose, two mice from each treatment group were sacrificed and their tumors were isolated. A significant increase in *BRRIAR* and ISG expression was confirmed in LNP-*BRRIAR*-treated tumors compared to LNP-control-treated tumors (Fig. 4j). Two days after the final dose, two mice per treatment group were sacrificed for tumor and organ isolation, showing higher *BRRIAR* expression in the tumors, spleens and livers of the LNP-*BRRIAR* group compared to controls (Fig. 4k). While *BRRIAR*-LNP-treated tumors showed no change in Ki67 + staining, they exhibited increased tumor cell death as indicated by TUNEL analysis (Supplementary Fig. 6j). To evaluate the effects of *BRRIAR*-LNPs on tumor growth, mice were imaged two days after treatment completion. Tumors treated with *BRRIAR*-LNPs showed reduced growth (6/7 mice), whereas tumors treated with empty-LNPs continued to grow (6/7 mice; Fig. 4l, m and Supplementary Fig. 6k). Although *BRRIAR*-LNP-treated tumors resumed growth ~ 30 days post-treatment, their progression was substantially slower than that of tumors treated with empty LNPs (Fig. 4m), resulting in extended survival (Fig. 4n). Treated mice maintained their weight and exhibited no signs of distress following treatment (Supplementary Fig. 6l). These findings demonstrate that IVT *BRRIAR* induces RIG-I-mediated apoptosis in ER + breast cancer cells in a cap-independent, dose-dependent manner, both *in vitro* and *in vivo* . 

### *BRRIAR*-induced cell death is selective for ER + breast cancer cells

**Fig. 5:**
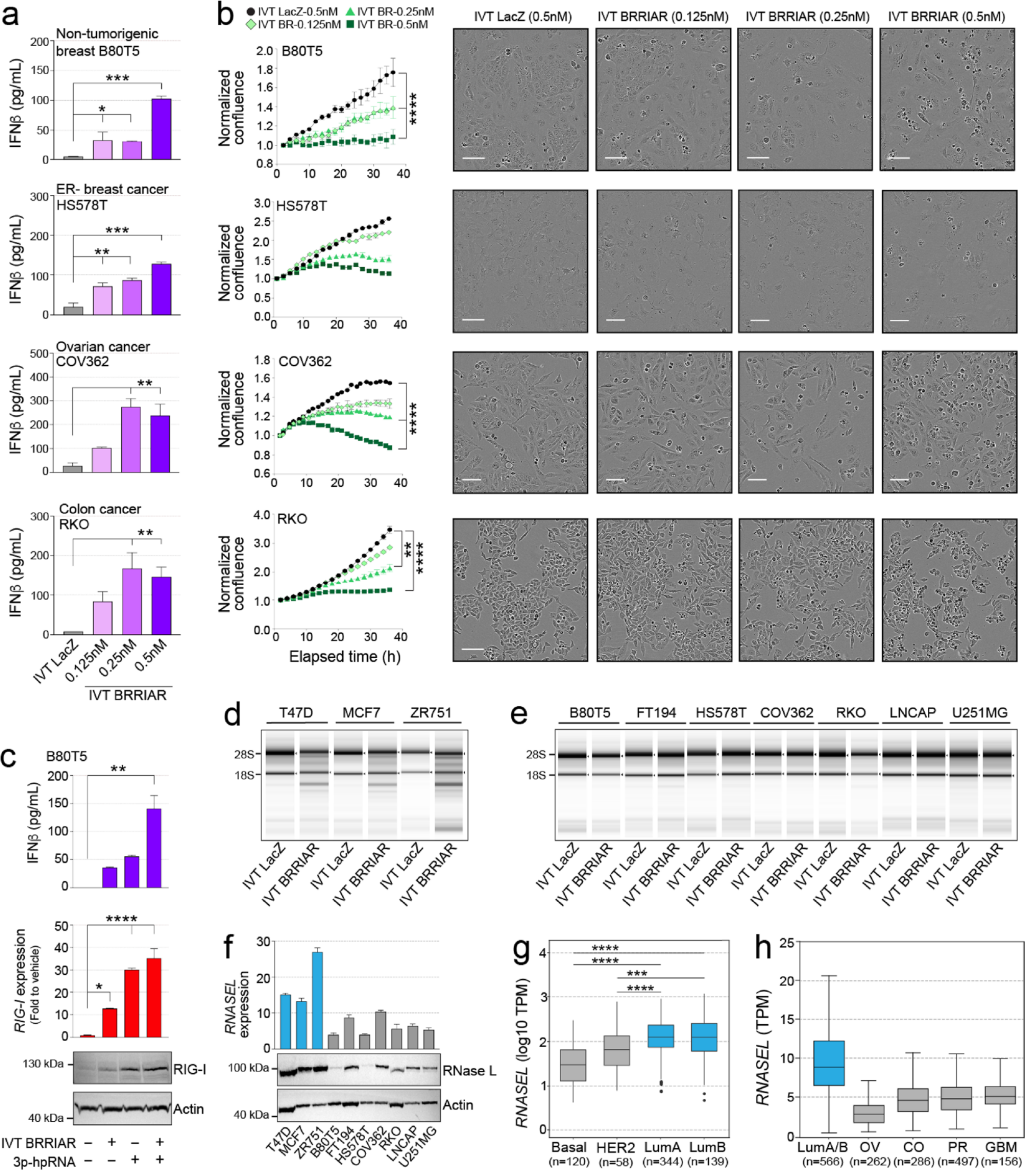
RNase L expression contributes to *BRRIAR-*mediated breast cancer cell death.** a** ELISA for IFNβ secreted from cells after transfection of IVT *LacZ* or IVT *BRRIAR*. Error bars, SD (*n* = 4). *p* values were determined by one-way ANOVA with Dunnett’s test (*p < 0.05, **p < 0.01, ***p < 0.001). **b** Left panels: cell confluence in cells measured by IncuCyte after transfection of IVT *LacZ* (0.5 nM) or IVT *BRRIAR* (0.125 nM, 0.25 nM, 0.5 nM). Error bars, SEM (*n* = 4). *p* values were determined by one-way ANOVA with Dunnett’s test (**p < 0.01, ****p < 0.0001). Right panels: representative IncuCyte images. Scale bar, 100 μm. **c** ELISA for IFNβ, qPCR and Western blot for RIG-I in B80T5 cells after transfection of 3p-hpRNA (0.5 µg/ml) and/or IVT *BRRIAR* (0.5 nM) for 6 h. *EIF2B1* was used as the qPCR internal control. Error bars, SEM (*n* = 3). *p* values were determined by one-way ANOVA with Dunnett’s test (*p < 0.05, **p < 0.01, ****p < 0.0001). Actin was used as the Western loading control. **d-e** TapeStation rRNA integrity analysis of total RNA isolated from **d** ER + breast cancer cells or **e** other cell types after transfection of IVT *LacZ* or IVT *BRRIAR* (0.5 nM) for 24 h. **f** qPCR and Western blot for RNase L in a panel of cancer cell lines. *EIF2B1* was used as the qPCR internal control. Actin was used as the Western loading control. **g** Boxplot of log10 *RNASEL* TPM (transcripts per million) in breast tumors from TCGA stratified by tumor subtype. *p* values were determined by Student’s *t-*test (***p < 0.001, ****p < 0.0001). **h** Boxplot of *RNASEL* TPM in additional tumor samples using the UALCAN web-based platform [31]. LumA/B = breast cancer (luminal A/B); OV = ovarian serous adenocarcinoma; CO = colon adenocarcinoma; PR = prostate carcinoma; GBM = glioblastoma multiforme.

We next examined whether IVT *BRRIAR*/RIG-I-mediated cell death was specific to ER + breast cancer cells or also occurred in non-tumorigenic cells and other cancer types. We first screened a panel of cell lines for functional RIG-I signaling by treating them with 3p-hpRNA and measuring RIG-I expression and IFNβ production. This analysis confirmed RIG-I activation in two non-tumorigenic cell lines, one ER- breast cancer and six additional cancer types (Supplementary Fig. 7a, b). Among these, ARK1 (endometrial cancer) was the only cell line that showed no IFNβ production after IVT *BRRIAR* transfection (Supplementary Fig. 7c, d). The remaining non-tumorigenic and cancer cell lines produced significantly higher levels of IFNβ after IVT *BRRIAR* transfection compared to the IVT *LacZ* control (Fig. 5a and Supplementary Fig. 7c, d), confirming *BRRIAR*-mediated RIG-I activation. IVT *BRRIAR* inhibited cell proliferation in a dose-dependent manner but did not induce cell death in most non-ER + breast cancer cell lines until the concentration reached at least 0.5 nM (Fig. 5b and Supplementary Fig. 7e). The exception was U251MG, the only non-breast cell line with low endogenous *BRRIAR* expression (Supplementary Fig. 1i), which also showed cell death at 0.25 nM of IVT *BRRIAR* (Supplementary Fig. 7e).

Multiple factors likely contribute to the selective induction of cell death by IVT *BRRIAR* in ER + breast cancer cells. One explanation is that *BRRIAR* acts as a primer for RIG-I activation rather than serving as a classical dsRNA ligand. To explore this in non-tumorigenic lines, which are expected to express minimal or no endogenous RIG-I ligands under normal conditions, we treated B80T5 breast cells with IVT *BRRIAR*, 3p-hpRNA or a combination of both. As expected, 3p-hpRNA treatment alone upregulated RIG-I expression and induced IFNβ production. In contrast, IVT *BRRIAR* alone modestly increased RIG-I protein expression and IFNβ production. Notably, co-treatment with both agents resulted in a robust enhancement of RIG-I expression and downstream pathway activation compared with 3p-hpRNA alone (Fig. 5c and Supplementary Fig. 7f). These results suggest that *BRRIAR* functions as a RIG-I primer, cooperating with classical RIG-I ligands to amplify signaling. Alternatively, the ER + breast tumor-specific apoptotic response may result from suboptimal activation of apoptosis pathways by IVT *BRRIAR* in non-ER + cells. A recent study reported that 3p-hpRNA-induced apoptosis requires both RIG-I-mediated priming and the 2’−5’ oligoadenylate synthetase (OAS)/RNase L system for execution [30]. To determine if this pathway contributes to IVT *BRRIAR*-induced cell death, we analyzed RNA integrity in ER + breast cancer cells following transfection. Cells treated with IVT *BRRIAR* showed ribosomal RNA (rRNA) degradation with a fragmentation pattern characteristic of RNase L activation (Fig. 5d). Furthermore, silencing *RNASEL* using dsiRNAs in T47D cells prior to IVT *BRRIAR* treatment partially abrogated the apoptotic

phenotype (Supplementary Fig. 7g, h). In contrast to ER + breast cancer cells, IVT *BRRIAR* alone did not activate RNase L-mediated apoptosis in our panel of non-tumorigenic and cancer cell lines (Fig. 5e). We confirmed the functionality of the OAS/RNase L pathway in these additional cell lines by treating them with high-dose polyI: C, a synthetic dsRNA analog that strongly induces RNase L activity (Supplementary Fig. 7i). Given that baseline RNase L levels have been reported to influence apoptosis sensitivity [32], we assessed its expression across our panel and found that ER + breast cancer cells expressed higher levels compared to all other tested cell lines (Fig. 5f and Supplementary Fig. 7j). Consistent with these findings, analysis of TCGA RNAseq data [31] revealed elevated *RNASEL* expression in ER + luminal breast tumors relative to other breast tumor subtypes (Fig. 5g) and additional selected tumor types (Fig. 5h).

### *BRRIAR *is primarily expressed in ER + breast tumor epithelial cells

**Fig. 6:**
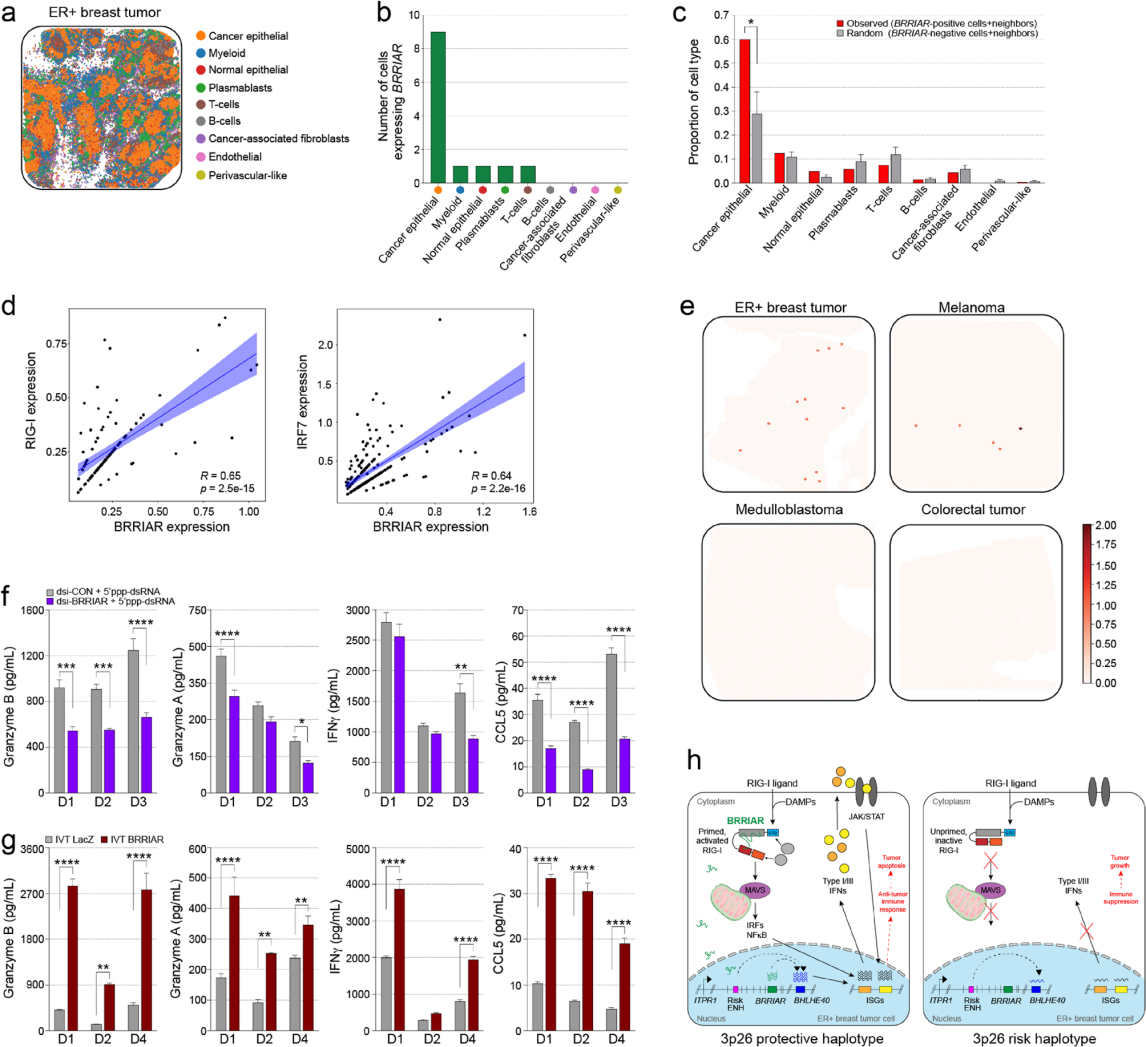
*BRRIAR* expression and immune activation in patient-derived samples.** a** High-resolution spatial transcriptomics map of one ER + breast tumor generated using the Curio Seeker platform. **b** Corresponding *BRRIAR*-positive cell states. **c** Spatial neighborhood analysis of *BRRIAR*-positive cells. Bars represent the proportion of neighbouring cell types within a defined radius. Error bars, SD (*n* = 13 cells). *p* value was determined by Student’s *t-*test (*p < 0.05). **d** Scatter plot showing correlation between *BRRIAR* and *RIG-I* (left panel) or *IRF7* (right panel) expression. Each black dot represents an individual cell at the co-expression peak. A linear regression line with its 95% confidence interval (blue shaded area) is overlaid. The correlation coefficient was calculated using Pearson correlation (*R*), and *p* values were determined using a two-sided *t*-test. **e** Spatially resolved *BRRIAR* expression across different tumor types using both 10x Visium and Curio Seeker platforms. *BRRIAR*-positive cells are depicted as colored dots. **f-g** Cytokine profiling for granzyme B, granzyme A, IFNγ and CCL5 secreted from HCMV-activated PBMCs (donors; D1-4) after **f** 48 h exposure to media harvested from T47D cells transfected with dsi-CON or dsi-*BRRIAR* then treated with 5’ppp-dsRNA for 3 h. The dsi-CON is a non-targeting control. Error bars, SEM (*n* = 4). Or **g** 48 h exposure to media harvested from T47D cells transfected with IVT *LacZ* or IVT *BRRIAR* for 24 h. Error bars, SEM (*n* = 4). *p* values were determined by two-way ANOVA with Sidak’s test (*p < 0.05, **p < 0.01, ***p < 0.001, ****p < 0.0001). **h** Proposed model for *BRRIAR* as a positive regulator of RIG-I activation in ER + breast tumor cells to promote IFN production and support an anti-tumor immune response

To assess the cell-type and tumor-type specificity of *BRRIAR* expression in patient-derived samples, we integrated spatial transcriptomic and scRNAseq data across multiple tumors. Consistent with our *in vitro* findings, spatial data showed that *BRRIAR* expression was predominantly localized to ER + breast tumor epithelial cells profiled with Curio Seeker (Fig. 6a, b**)**. Although limited by cell numbers, spatial neighborhood analysis revealed that *BRRIAR*-positive cells were surrounded by significantly more cancer epithelial cells (Fig. 6c and Supplementary Fig. 8a). In the spatial data, too few cells co-expressed *BRRIAR* with *RIGI* or *IRF7* to compute a meaningful correlation. However, analysis of scRNAseq breast tumor data (Fig. 1f) showed strong positive correlations between *BRRIAR* and *RIG-I* (Spearman’s *r* = 0.65, *p* = 2.4e-15) or *IRF7* (Spearman’s *r* = 0.64, *p* = 2.2e-16; Fig. 6d), suggesting elevated RIG-I signaling in *BRRIAR*-positive cells. To evaluate tissue specificity we analyzed additional spatial datasets, including another ER + breast tumor [33], a medulloblastoma PDOX-mouse model [34], a colorectal tumor [35] (all sequenced on 10x Visium), and a melanoma sample profiled with Curio Seeker. *BRRIAR* expression was highest in the ER + breast tumor sample, lower in melanoma and undetectable in the other tumor types (Fig. 6e). Similarly, analysis of scRNAseq datasets from five tumors showed highest expression in the ER + breast tumor, low expression in non-small cell lung cancer, and no expression in the remaining tumor types (Supplementary Fig. 8b).

### *BRRIAR* stimulates IFN-mediated immune responses in human PBMCs

ER + breast tumors often establish an immunosuppressive tumor microenvironment (TME) with low levels of tumor-infiltrating lymphocytes (TILs) and PD-L1, making them resistant to PD-1 therapies [36, 37]. As reduced *BRRIAR* expression is associated with breast cancer risk variants, we hypothesized that lower *BRRIAR* levels impair RIG-I activation and contribute to immune suppression, although this effect would likely be limited to a small subset of cells. To address this, we examined whether *BRRIAR* knockdown in breast cancer cells could affect cytokine production by activated human immune cells. To generate an antigen specific immune response, independent of immune suppression during cancer, PBMCs from healthy donors with a previous HCMV infection were stimulated with HCMV peptides and cultured with conditioned media collected from T47D dsi-CON or dsi-*BRRIAR* cells treated with 5’ppp-dsRNA. Cytokine profiling showed that media from T47D dsi-*BRRIAR* cells significantly reduced the release of key pro-inflammatory cytokines, including granzyme B, granzyme A, IFNγ and CCL5, after incubation with PBMCs compared to media from T47D dsi-CON cells (Fig. 6f). Conversely, conditioned media from T47D cells transfected with IVT *BRRIAR* induced robust cytokine secretion compared to IVT *LacZ*-treated cells (Fig. 6g and Supplementary Fig. 8c), highlighting the immunostimulatory potential of *BRRIAR*. Given that cytotoxic CD8 + T cells and natural killer (NK) cells are the primary tumoricidal effector cells responsible for generating granzymes, we performed flow cytometry on cultured cells to confirm these populations as the source of granzyme B (Supplementary Fig. 8d). Using media from dsi-CON cells, PBMCs labelled with a multi-antibody panel, confirmed that granzyme B was produced by CD8 + T cells and NK cells (Supplementary Fig. 8e). These findings suggest that *BRRIAR* can promote pro-inflammatory cytokine production during an antigen-specific (HCMV) immune response and may potentiate CD8 + T cell and NK cell-mediated tumor cell killing in ER + breast cancer.

## Discussion

GWAS have identified thousands of genetic variants associated with complex diseases but connecting the variants to their causal genes remains a major challenge. In this study, we identify a recently annotated lncRNA, *BRRIAR* as a key target gene at the 3p26 breast cancer risk locus. Functional studies showed that *BRRIAR* regulates *BHLHE40 in cis*, and transcriptomic profiling of ER + breast cancer cells following knockdown of either *BRRIAR* or *BHLHE40* revealed a shared downregulation of ISGs, implicating both genes as modulators of IFN signaling. *BHLHE40* encodes a TF with well-established roles in cellular differentiation, circadium rhythm and immune regulation [38]. Its loss has been linked to impaired cytokine signaling and T cell exhaustion, particularly during chronic infection and autoimmunity [39]. A striking feature of *BRRIAR* is its dual functionality depending on subcellular localization, a characteristic shared by only a few lncRNAs. For example, *PYCARD-AS1* localizes to the *PYCARD* promoter to facilitate DNA methylation while also binding to *PYCARD* mRNA in the cytoplasm to inhibit translation [40]. Although *BRRIAR* did not affect the expression of other nearby genes, it is notable that the region harbors at least two other lncRNAs with immunomodulatory roles. *BHLHE40-AS1*, which lies antisense to the *BHLHE40* promoter, functions independently of BHLHE40 and has been shown to modulate IL-6/STAT3 signaling in early-stage breast cancer, contributing to an immunosuppressive TME that promotes tumor progression [41]. Another lncRNA, *EGOT* (eosinophil granule ontogeny transcript), located ~ 36 kb from *BRRIAR*, is induced by cellular stress, viral infection or PAMPs, particularly in liver and kidney cells [42]. Depending on the cellular context, *EGOT* can repress or activate ISG transcription to regulate antiviral responses. Together, these findings position 3p26 as a regulatory hotspot for lncRNAs involved in antiviral and immune signaling. 

*BRRIAR* is an ESR1-dependent lncRNA transcribed from an ~ 11 kb enhancer cluster, although our data suggest that estrogen acts indirectly. Silencing the *BRRIAR* promoter not only reduces its expression but also diminishes enhancer cluster activity, leading to downregulation of *BHLHE40*. This suggests that *BRRIAR* transcription is required for the full enhancer activity in a subset of ER + breast tumors. While the precise mechanistic step at which *BRRIAR* acts remains unclear, continuous transcription of eRNAs and lncRNAs through enhancers is known to sustain chromatin accessibility and histone acetylation at defined regulatory regions [43]. Consistent with this, several RNA-binding proteins were identified in the *BRRIAR* IP-mass spectrometry analysis, with hnRNPF as the top hit. HnRNPF is known to associate with pre-mRNAs and regulate multiple aspects of post-transcriptional processing. We deprioritised hnRNPF, as it was difficult to determine whether it affects *BRRIAR* RNA processing rather than mediating its function. Of note, hnRNPF was recently shown to bind an eRNA at the lactotransferrin enhancer to facilitate enhancer-promoter chromatin looping [44], raising the possibility that hnRNPF binding to *BRRIAR* could similarly support enhancer-promoter communication. Beyond transcriptional regulation, the *BRRIAR* RNA transcript contributes to chromatin looping at the enhancer cluster to regulate *BHLHE40*, a mechanism shared by several other lncRNAs involved in enhancer-promoter interactions [45]. Collectively, our findings contribute to the growing body of evidence that elncRNAs are not just transcriptional byproducts but are required for enhancer function.

Once exported from the nucleus, cytoplasmic *BRRIAR* binds to the PRR RIG-I, modulating both its expression and activation. Silencing cytoplasmic *BRRIAR* reduced RIG-I activation in response to the synthetic ligand 3p-hpRNA, indicating a role in priming the receptor rather than serving as a ligand itself. This is supported by the observation that *BRRIAR* does not bind to the CTD of RIG-I, distinguishing it from canonical 5’PP/PPP viral ligands. Further supporting this model, *BRRIAR* and 3p-hpRNA exhibit functional synergy. Co-delivery of IVT-*BRRIAR* and 3p-hpRNA into non-tumorigenic breast cells led to a synergistic increase in both RIG-I expression and IFNβ production, consistent with *BRRIAR* enhancing the responsiveness to a canonical ligand. In contrast, IVT-*BRRIAR* alone did not elevate RIG-I levels, whereas 3p-hpRNA alone increased both RIG-I and IFNβ, reflecting full pathway activation through positive feedback. In cancer cells, RIG-I can be aberrantly activated by endogenous RNAs arising from dysregulated RNA processing or stress-induced RNA release. We propose that in ER + breast cancers, the presence of *BRRIAR* sensitizes the RIG-I pathway, thereby amplifying innate immune and anti-proliferative responses. The breast cancer risk region at 3p26, where the risk haplotype reduces *BRRIAR* expression, may therefore contribute to tumorigenesis by dampening this pathway.

The mechanism by which *BRRIAR* primes RIG-I remains unclear, however it is possible that *BRRIAR* stabilizes RIG-I’s active conformation or facilitates its oligomerization by recruiting co-factors essential for its phosphorylation or ubiquitination. While no human lncRNA has yet been shown to function this way, such a mechanism would be analogous to the activity of mouse lncRNA *Lnczc3h7a* [14] or protein regulators such as TRIM25 [46] and RIPLET [47]. Alternatively, *BRRIAR* may act by destabilizing intramolecular contacts that maintain RIG-I in its autoinhibited conformation [11], thereby lowering the energy threshold for ATP-driven hinge opening and allowing activation by low abundant or suboptimal ligands. This model is supported by *BRRIAR’s* binding with the CARD and helicase domains, regions critical for maintaining RIG-I in an inactive state. Another possibility is that *BRRIAR* made be required as a seeding agent for RIG-I phase separation, promoting the local assembly of RIG-I and its cofactors. Other lncRNAs, such as *NEAT1* [48] and *NORAD* [49], are known to facilitate the formation of paraspeckles and stress granules through similar mechanisms.

We present genetic and functional evidence implicating *BRRIAR* in the pathogenesis of ER + breast cancer and provide proof-of-concept that IVT *BRRIAR* may serve as an effective RNA therapeutic. IVT *BRRIAR* exhibits dual activity, selectively inducing intrinsic apoptosis in tumor cells while concurrently activating both innate and adaptive anti-tumor immune responses. However, IVT *BRRIAR* levels greatly exceed physiological levels and remain cytoplasmic, meaning this approach does not recapitulate ESR1- or BHLHE40-dependent regulation, nor the local enhancer effects reported for other elncRNAs. Supporting *BRRIAR’s* broader therapeutic relevance, ATACseq data show that the *BRRIAR*-associated enhancer cluster is active in a subset of other tumor types. Importantly, only *BRRIAR*-expressing cell lines undergo apoptosis in response to low-dose IVT *BRRIAR*, underscoring its potential as both a predictive biomarker and a tumor-selective therapy. This is significant given the growing interest in RIG-I agonists as anti-cancer agents. In preclinical models, RIG-I activation reduces tumor burden and boosts TIL recruitment including in triple-negative breast cancer [13]. Clinical trials are currently evaluating synthetic RIG-I agonists (e.g. SLR14 [50], MK-4621 [51]) as monotherapies or in combination with immune checkpoint inhibitors (ICIs). While well-tolerated, their anti-tumor activity have been modest, likely due to suboptimal delivery or lack of tumor-specific activation. To address these limitations, delivery methods such as red blood cell-derived vesicles [52] or antibody-RNA conjugates [53] could be adapted for *BRRIAR*, expanding the clinical utility of RIG-I-based therapies.

This study has several limitations. First, *BRRIAR* is not conserved in mice, necessitating the use of human ER + breast cancer cells xenografted into immunocompromised murine hosts to evaluate the intrinsic effects of IVT *BRRIAR* on tumor cell apoptosis. While this model allowed assessment of *BRRIAR*’s proinflammatory effects, we were unable to test its impact on the immune response. Although RIG-I agonists have shown synergy with ICIs, the absence of a competent immune system in our model precludes preclinical testing of IVT *BRRIAR* in this therapeutic context. Future studies will require the use of humanized mouse models to evaluate the additional benefits of IVT *BRRIAR* on the TME. This challenge highlights a broader issue in lncRNA research, as many therapeutically relevant lncRNAs are not conserved in standard animal models. Second, while our findings indicate that *BRRIAR* primes RIG-I activation, we have not yet demonstrated a direct physical interaction between *BRRIAR* and RIG-I. It remains plausible that *BRRIAR* engages an intermediary protein that subsequently activates RIG-I. Further studies, such as cryo-electron microscopy or fluorescence resonance energy transfer will be necessary to resolve the molecular details of the interaction.

## Conclusions

In summary, we propose a genetic and mechanistic model in which variants at 3p26 alter ER + breast cancer risk by regulating the expression of the *BRRIAR* lncRNA (Fig. 6h). Individuals carrying the protective 3p26 haplotype express *BRRIAR* in a subset of ER + breast tumors, where it maintains *BHLHE40* and ISG expression and primes RIG-I for activation. When RIG-I is activated by DAMPs, viral RNAs, or other ligands, *BRRIAR* enhances IFN signaling, the production of pro-inflammatory cytokines and an anti-tumor immune response. Conversely, individuals with the risk-associated 3p26 haplotype have absent or reduced expression of *BRRIAR* and *BHLHE40* in a subset of ER + breast tumors, resulting in attenuated ISG transcription and increased tumor cell proliferation. In parallel, lower *BRRIAR* levels impair RIG-I activation and IFN signaling, suppressing pro-inflammatory cytokine production and fostering a more immunosuppressive TME that favors ER + breast tumor development. These findings highlight IVT *BRRIAR* as a promising therapeutic candidate to restore innate immune sensitivity and restrict ER + breast tumor growth.

## Methods

Catalog numbers for all kits and reagents are provided in Supplementary Table 16.

### Cell lines and culture

T47D, MCF7, BT474 and BT549 (ATCC) cells were grown in RPMI 1640 medium (Thermo Fisher Scientific) with 10% fetal bovine serum (FBS; Thermo Fisher Scientific), 1 mM sodium pyruvate (Thermo Fisher Scientific), 10 µg/ml insulin (Thermo Fisher Scientific), and 1% antibiotics (Thermo Fisher Scientific). MDAMB361 and MDAMB134VI (ATCC) were grown in DMEM medium (Thermo Fisher Scientific) with 20% FBS and 1% antibiotics. SUM149 (Applied Biological Materials), BT20, RKO, PANC1 (ATCC) and COV362 (Merck) were grown in DMEM medium with 10% FBS and 1% antibiotics. B80T5 (kindly provided by Prof R Reddel, CMRI, AUS [54]), ARK1 (kindly provided by Prof AD Santin, UAMS, USA [55]), HS578T, ZR751, MDAMB468, MDAMB231, LNCAP, HEK293 (ATCC) and U251MG (Merck) were grown in RPMI 1640 medium with 10% FBS and 1% antibiotics. MCF10A (ATCC) were grown in DMEM/F12 medium (Thermo Fisher Scientific) with 5% horse serum (Thermo Fisher Scientific), 10 µg/ml insulin, 0.5 µg/ml hydrocortisone (Merck), 20 ng/ml epidermal growth factor (Thermo Fisher Scientific), 100 ng/ml cholera toxin (Merck), and 1% antibiotics. HMEC and 184A1 (ATCC) were grown in MEBM basal medium supplemented with MEGM SingleQuots (Lonza). FT194 (ATCC) were grown in DMEM/F12 medium with 10% FBS and 1% antibiotics. T47D-TamR and MCF7-TamR (ATCC) were grown in T47D medium (as described above) supplemented with 1 µM 4-hydroxytamoxifen (Merck). MCF7-cTamPalbR cells (resistant to tamoxifen and palbociclib) were developed by growing parental MCF7 cells (Michigan Cancer Foundation) in phenol red-free RPMI 1640 with 5% charcoal-stripped FBS (CS-FBS; Thermo Fisher Scientific), 1% antibiotics and 10 pM 17β-estradiol (Merck, resuspended in ethanol). Resistance to tamoxifen and palbociclib was developed by continuous culture in 500 nM 4-hydroxytamoxifen (Merck; resuspended in tetrahydrofuran) and 250 nM palbociclib (Selleckchem; resuspended in water) over 16 months, after which cells were maintained with the same drug concentrations. Passage-matched MCF-7 vehicle cells were maintained with tetrahydrofuran (Merck). Cell lines were maintained under standard conditions (37 °C, 5% CO_2_), tested for *Mycoplasma* and profiled for short tandem repeats.

### TCGA datasets and analyses

*BRRIAR* expression was initially quantified in TCGA RNAseq data, and eQTL analysis performed as described in Moradi-Marjaneh et al. [4]. For this study, gene-level normalized expression for *BRRIAR*, *RIG-I/DDX58*,* RNASEL* and *BHLHE40* was analyzed across TCGA breast tumor subtypes, with subtype labels obtained from Netanely et al. [56]. Expression values were log-transformed, visualised as boxplots with sample counts and compared between subtypes using Welch’s two-sample t-tests. Analyses and visualizations were performed in R and scripts are available on request. For tumor purity analysis, consensus purity estimates were from Aran et al. [57] were matched to TCGA breast tumor using sample barcodes. Associations between *BRRIAR* expression and tumor purity were evaluated using Pearson correlation. PAM50 subtypes were obtained from Thorsson et al. [58]. For ATACseq analysis, data generated by Corces et al. [23] for the hg38 region (chr3:4681163–4726446) were downloaded from the UCSC Xena Browser. Breast cancer-specific regions of open chromatin were identified in TCGA BRCA samples [23], and PAM50 intrinsic subtypes were assigned using the GDC TCGA breast cancer release [59]. The ATACseq signal was expressed as quantile-normalized, log2-transformed counts per million, and visualized using the *pheatmap* R package.

### Quantitative real-time PCR (qPCR)

Total RNA was extracted using TRIzol (Thermo Fisher Scientific). Complementary DNA (cDNA) was synthesized using SuperScript IV (Thermo Fisher Scientific) and random primers, except for hnRNA detection where a gene-specific primer was used. qPCR was performed using TaqMan assays (Thermo Fisher Scientific) as per the manufacturer’s protocol, or with Syto9 incorporation into PCR-amplified products using gene-specific primers (Integrated DNA Technologies; IDT) on a Mic qPCR machine (Bio Molecular Systems). *GAPDH* (glyceraldehyde-3-phosphate dehydrogenase) or *EIF2B1* (eukaryotic translation initiation factor 2B subunit alpha) served as internal reference controls, and relative expression was calculated using the delta Cq method. Taqman assays and primer sequences are listed in Supplementary Table 17.

### Estrogen and tamoxifen treatments

T47D cells were cultured in phenol red-free RPMI 1640 media with 5% CS-FBS and 1% antibiotics for 48 h, then treated with DMSO (vehicle control; Merck), 17β-estradiol (10 nM; Merck), 4-hydroxytamoxifen (1 µM; Merck) or their combination for 0, 2, 6 and 24 h. Cells were harvested with TRIzol, and estrogen and tamoxifen responses evaluated by qPCR of *GREB1* (growth regulating estrogen receptor binding 1), an established estrogen-responsive target gene.

### Cell fractionation

T47D and ZR751 cells were fractionated using the PARIS kit (Thermo Fisher Scientific) as per the manufacturer’s protocol. Briefly, 2 × 10^6^ cells per replicate were harvested, washed, pelleted, and resuspended in cell fractionation buffer. After incubation on ice, samples were centrifuged to separate the cytoplasmic fraction (supernatant) from the intact nuclear pellet. The nuclear pellet was then lysed in cell disruption buffer and RNA from both cytoplasmic and nuclear fractions was purified using the silica column-based protocol supplied with the kit. Fraction purity was assessed by qPCR using *NEAT1* (nuclear enriched abundant transcript 1) and *GAPDH* as positive controls for nuclear and cytoplasmic RNA, respectively. As mature *BRRIAR* RNA was not reliably detected in nuclear fractions, *hnBRRIAR* and *hnGAPDH* were quantified using gene-specific cDNA and qPCR primers. Primers are listed in Supplementary Table 17.

### Random amplification of cDNA ends (RACE)

5’ and 3’ RACE were performed on 5 µg of total RNA from T47D cells using the GeneRacer kit (Thermo Fisher Scientific) as per the manufacturer’s protocol. cDNA synthesis and RACE amplification were performed using the kit adapters and *BRRIAR*-specific primers (listed in Supplementary Table 17). Amplified products were resolved on 2% agarose gels before being purified with the Qiaquick Gel Extraction Kit (Qiagen). Purified amplicons were cloned using the Zero Blunt PCR cloning kit (Thermo Fisher Scientific) and Sanger sequenced at the Australian Genome Research Facility (AGRF).

### Cell proliferation assay

Cell proliferation was measured using the IncuCyte live-cell imaging system (Essen Bioscience). Cells were seeded at 2–4 × 10^4^ cells per well into 24-well plates and imaged using a 10x objective lens every 3 h over 1–7 days. Imaging was performed in an incubator maintained at 37 °C under a 5% CO_2_. Cell confluence was measured using IncuCyte ZOOM 2016 A software and the data analyzed using GraphPad Prism.

### Western blotting

Cell pellets were lysed in RIPA buffer (50 mM Tris-HCl, pH 8.0, 150 mM NaCl, 1% IGEPAL CA-630, 0.5% sodium deoxycholate, 0.1% sodium dodecyl sulfate (SDS), 1 mM DTT, protease inhibitor cocktail (PIC; Roche) and centrifuged to remove cell debris. Proteins were separated by SDS-polyacrylamide gel electrophoresis, electroblotted onto PVDF membranes by semi-dry transfer (Bio-Rad) and blocked in 1% casein blocking buffer (Bio-Rad). Antibodies detecting BHLHE40 (1:2000; Novus), RIG-I (1:2000; Abclonal), Caspase-3 (1:1000; Cell Signaling), RNase L (1:1000; Cell Signaling), β-Actin (1:20,000; Cell Signaling) or FLAG (1:2000; Cell Signaling) were incubated 4 °C 16 h. Horseradish peroxidase (HRP) secondary antibodies were used for detection (Cell Signaling). Proteins were visualized with enhanced chemiluminescence substrate (Pierce) and the iBright imaging system (Thermo Fisher Scientific).

### RNA-fluorescence in situ hybridization (FISH) and Immunofluorescence (IF)

T47D cells grown on coverslips were fixed with Image-iT fixative solution (Thermo Fisher Scientific) and permeabilized with 70% ethanol before being neutralized with 125 mM glycine (Merck). For RNA-FISH, cells were stained for 16 h with 125 nM of a custom *BRRIAR* Stellaris RNA-FISH probe set labelled with Quasar 570 fluorophore (Biosearch Technologies) as per the manufacturer’s protocol. For IF staining, cells were re-permeabilized with 0.5% Triton X-100 before incubation with rabbit anti-RIG-I (1:100, Abclonal) and anti-rabbit Alexa Fluor Plus 488 antibodies (1:500, Thermo Fisher Scientific). Coverslips were mounted onto slides using ProLong Glass antifade medium containing NucBlue counterstain (Thermo Fisher Scientific). Multiple fields were imaged on a DeltaVision personalDV microscope using a 100x objective lens in the FITC, TRITC and DAPI channels. Z-stacks were deconvolved and projected using Softworx software (GE Healthcare) and subcellular puncta were quantified using Imaris software (Oxford Instruments).

### Chromosome conformation capture (3C)

3 C libraries were generated using *Eco*RI as described previously [60]. 3 C interactions were quantified by qPCR using primers designed within restriction fragments (listed in Supplementary Table S17). qPCR was performed on a RotorGene 6000 using MyTaq HS DNA polymerase (Bioline) with the addition of 25 µM SYTO9, annealing temperature of 66 °C. 3 C analyses were performed on two independent 3 C libraries from each cell line quantified in duplicate. Bacterial artificial chromosome (BAC) clones spanning each region were used to generate artificial libraries of ligation products to normalize for PCR efficiency. Data were normalized to the signal from the BAC clone library and, between cell lines, by reference to a region within *GAPDH*.

### Chromatin immunoprecipitation (ChIP)

T47D cells were cross-linked with 1% formaldehyde (Merck) at 37 °C for 10 min, rinsed with phosphate buffered saline (PBS; Thermo Fisher Scientific) containing 5% bovine serum albumin (BSA; Scientifix) and harvested in PBS containing PIC. Harvested cells were centrifuged for 2 min at 3,000 rpm. Cell pellets were resuspended in lysis buffer (1% SDS, 10 mM EDTA, 50 mM Tris-HCl, pH 8.1, PIC) and sonicated three times for 15 s at 70% duty cycle (Branson SLPt) then centifuged at 13,000 rpm for 15 min. Supernatants were resuspended in dilution buffer (1% Triton X-100, 2 mM EDTA, 150 mM NaCl, 20 mM Tris-HCl, pH 8.1). Antibodies detecting H3K27ac (2 µg; Abcam) or an IgG control (2 µg; Abcam) were prebound for 6 h to protein G Dynabeads (Thermo Fisher Scientific), then incubated with the chromatin for 16 h. The complexes were then washed six times in RIPA buffer. To reverse cross-links, the complexes were incubated at 65 °C for 16 h in elution buffer (1% SDS, 0.1 M NaHCO_3_), then DNA fragments purified using the QIAquick spin kit (Qiagen). For ChIP-qPCR, 2 µl of chromatin extract were amplified with primers spanning the 11 kb enhancer cluster on a RotorGene 6000 using MyTaq HS DNA polymerase (Bioline) with the addition of 25 µM SYTO9, annealing temperature of 60 °C (primers listed in Supplementary Table 17). For ChIPseq, libraries were prepared using the NEBNext Ultra II DNA library prep kit (New England Biolabs; NEB), according to the manufacturer’s instructions. Samples were barcoded and run on an Illumina NovoSeq 6000 platform.

### H3K27ac ChIPseq analysis

Raw sequencing data were checked for quality using FastQC v0.11.5 and adapters trimmed using Cutadapt v1.13 [61]. Paired-end reads were aligned to the hg19 human reference genome using Bowtie2 v2.2.9 [62] and filtered using PicardTools v2.19.0. MACS2 v2.1.2 [63] was used to call peaks representing regions of enriched H3K27ac signal. Global signal tracks were generated with bamCompare (deepTools) [64] and visualized with the Integrative Genomics Viewer (IGV) browser. Enriched peaks were assigned to genes within a 5 kb linear distance and over-represented pathway terms were determined using the ReactomePA Bioconductor package v1.9.4 [65]. To assess the relationship between enhancer activity and gene expression, H3K27ac ChIP-seq changes were compared to RNA-seq expression changes. Each gene was assigned the nearest H3K27ac peak within 5 kb of the transcription start site, and log fold changes were calculated relative to control non-targeting guides. Scatter plots and linear regression were generated in R (v4.2.0), with Pearson correlation coefficients and *p*-values calculated using the *ggpubr* package.

### RNA sequencing (RNAseq) analysis

Total RNA was extracted from cells using the RNeasy Plus mini kit (Qiagen). Libraries were prepared using the TruSeq stranded mRNA library prep kit (Illumina). Samples were barcoded and run on a NextSeq 550 (Illumina). Sequencing quality was assessed with FastQC. Paired-end reads were trimmed with Trimmomatic [66] and aligned to the hg38 human reference genome using STAR [67]. SAMtools [68] and NovoSort were used to filter reads. Transcript quantification was performed with RSEM [69] to obtain transcripts per million (TPM) counts, which were summed to derive gene-level counts. EdgeR [70] was used to find differentially expressed genes (DEGs), and false discovery rates (FDR) were calculated using the Benjamini-Hochberg method. As *BRRIAR* regulates more restricted programs than *BHLHE40*, different DEG cutoffs were applied: *BRRIAR* (|logFC| >0.5, FDR < 1%) and *BHLHE40* (|logFC| >1.0, FDR < 1%). This adjustment produced DEG lists of comparable size while maintaining statistical stringency.

### RNA accessibility assays

T47D cell pellets were resuspended in lysis buffer (50mM Tris-HCl, pH8, 150mM NaCl, 0.5% NP-40, PIC), incubated at 4 °C for 30 min, then the volume adjusted to 1 ml with 1x RNaseH buffer (NEB). Lysates (100 µl) were mixed with 100 pmol DNA oligonucleotides and incubated at 4 °C for 2 h before adding 2.5 U RNaseH. Reactions were incubated at 37 °C for 20 min before RNA was isolated using TRIzol for qPCR (oligos and primers listed in Supplementary Table 17).

### Oligonucleotide transfections

Cells were transfected with 200 nM antisense oligonucleotides (ASO-CON negative control or *BRRIAR*-targeting ASOs, SynGenis; listed in Supplementary Table 17) or 100 nM Dicer-substrate small interfering RNAs (dsi-CON negative control or *BRRIAR*-targeting dsiRNAs, IDT; listed in Supplementary Table 17) using RNAiMAX (Thermo Fisher Scientific) as per the manufacturer’s protocol. Cells were harvested 48 h post-transfection for downstream functional assays.

### Plasmid construction

For IVT *BRRIAR*, full-length *BRRIAR* was synthesized as a gBlock (IDT) and cloned into pGEM-T (Promega). Individual CRISPRi gRNAs, spacers, Cas9 binding handle and terminator sequences were synthesized as gBlocks (IDT) and cloned into the lentiviral vector pgRNA-humanized (a gift from Stanley Qi [Addgene; #44248]). All constructs were Sanger sequenced at the AGRF.

### RNA* in vitro *transcription (IVT)

The pGEM-T-*BRRIAR* and pEF-ENTR-LacZ (a gift from Eric Campeau & Paul Kaufman [Addgene; #17430]) plasmids were linearized near the 3’ end by restriction digestion and IVT was performed using the HiScribe T7 Quick High Yield RNA synthesis kit (NEB) as per the manufacturer’s protocol. RNA was isolated using TRIzol and DNase treated using TURBO DNase (Thermo Fisher Scientific). The HiScribe T7 ARCA mRNA kit (NEB) was used to produce cap-0 IVT *BRRIAR* from the linearized pGEM-T plasmid, and further processed with mRNA Cap 2´-O-Methyltransferase (NEB) to produce cap-1 IVT *BRRIAR*. IVT RNA was transfected into cells using Lipofectamine 3000 (Thermo Fisher Scientific) and harvested at 6–40 h post-transfection.

### RNA-protein pull-down and mass spectrometry

IVT RNA (50 pmol) was 3’-end labelled using the Pierce RNA 3’ end desthiobiotinylation kit (Thermo Fisher Scientific) as per the manufacturer’s protocol. Labeled RNA was purified using the Direct-zol RNA miniprep kit (Zymo Research) and immobilized on streptavidin-coated magnetic beads (Pierce magnetic RNA-protein pull-down kit; Thermo Fisher Scientific). T47D cells grown in 10 cm dishes were trypsinized, washed with PBS and pelleted by centrifugation. Cell pellets were resuspended in lysis buffer (25 mM Tris-HCl, pH 7.4, 150 mM NaCl, 1 mM EDTA, 5% glycerol, 1% IGEPAL, 1x RNAsecure (Thermo Fisher Scientific), PIC) on ice for 10 min, then centrifuged. Supernatants were collected and protein concentrations determined by Bradford assay (Bio-Rad). For each pull-down, 200 µg of lysate were incubated with magnetic beads pre-bound with either IVT *BRRIAR* or IVT *LacZ* at 4 °C for 1 h. Beads were washed three times with wash buffer to remove unbound proteins, and three times with 20 mM Tris-HCl (pH 7.5) to remove excess detergent. Bound proteins were subsequently identified by mass spectrometry as previously described [7].

### RNA immunoprecipitation (RIP)

T47D cells were cross-linked with 1% formaldehyde at 37 °C for 10 min, quenched with 0.25 M glycine and centrifuged for 2 min at 100 x g. Cell pellets were resuspended in lysis buffer (50 mM HEPES, pH 7.5, 0.4 M NaCl, 1 mM EDTA, 1 mM DTT, 0.5% Triton X-100, 10% glycerol, 0.1 M phenylmethylsulfonyl fluoride (PMSF), 200 U RNase inhibitor (NEB), PIC), sonicated ten times for 10 s at 70% duty cycle (Branson SLPt) and clarified by centrifugation. For IP, anti-FLAG magnetic beads (Cell Signaling) were blocked in lysis buffer with 1% BSA at 4 °C 16 h, then incubated with lysates at 4 °C 16 h. The magnetic bead-protein/RNA complexes were collected and washed five times in wash buffer (50 mM HEPES, pH 7.5, 0.1 M NaCl, 5 mM EDTA, 10 mM DTT, 0.5% Triton X-100, 10% glycerol, 1% SDS, 200 U RNase inhibitor) and RNA was recovered by TRIzol extraction and treated with RNase-free DNaseI (NEB).

### Synthetic RIG-I agonist transfections

T47D cells were transfected with 3p-hpRNA (0.5 µg/ml; Invivogen) using Lipofectamine 3000, or 5’ppp-dsRNA (1 µg/ml) and its non-phosphorylated control dsRNA (Invivogen) using 6.25 µg of LyoVec transfection reagent (Invivogen) as per the manufacturer’s protocol. Cells were harvested 6–40 h post-transfection for downstream functional assays.

### ELISA (enzyme-linked immunosorbent assay)

The concentrations of human IFNβ and IFNλ1 (IL-29) in cell culture media were quantified using ELISA kits (Abcam) as per the manufacturer’s protocol. Briefly, 50 µl of the collected media were added to pre-coated 96-well plates and incubated with detection antibodies at 25 °C for 1 h. Plates were then washed before being developed with colorimetric substrate. Absorbance was measured at 450 nm using a Tecan i-control plate reader and concentrations calculated by interpolation using standard curves.

### Lentivirus production and transduction

Lenti-dCas9-KRAB-blast (a gift from Gary Hon [Addgene; #89567]), Lenti-Cas9‐2 A‐Blast plasmid (a gift from Jason Moffat [Addgene; #73310]), pgRNA-humanized (a gift from Stanley Qi [Addgene; #44248])or Human Brunello CRISPR knockout pooled library (a gift from David Root and John Doench [Addgene; #73178]) were co-transfected with two package plasmids, pMD2.G (a gift from Didier Trono [Addgene; #12259]) and psPAX2 (a gift from Didier Trono [Addgene; #12260]) into HEK293 cells using FuGENE6 (Promega). The culture supernatant containing virus-like particles (VLPs) was harvested after 24–48 h of incubation and filtered through a 0.45 μm membrane. The VLPs were concentrated by centrifugation at 10,000 rpm at 4 °C for 16–24 h, resuspended in RPMI 1640 medium supplemented with 10% FBS, aliquoted, and stored at −80 °C. For CRISPRi, cells were transduced by VLPs by directly addition. Two days post-transduction, cells were selected with 10 µg/ml blasticidin (Thermo Fisher Scientific) and 2 µg/ml puromycin (Thermo Fisher Scientific) for 10 days to kill non-transduced cells.

### IVT RNA and lentivirus titering

IVT *LacZ* and IVT *BRRIAR*: MCF7 cells (2.8 × 10^6^) were seeded into flasks before IVT *LacZ* or IVT *BRRIAR* were transfected using Lipofectamine 3000 at final concentrations of 0, 1, 5, 10, 25 or 50 pM. After 48 h, live cells in each flask were counted using a Countess II FL automated cell counter (Thermo Fisher Scientific). The IC_80_ values for IVT *BRRIAR* were calculated in Prism (GraphPad). Lenti-Cas9‐2 A‐Blast VLPs: MCF7 cells (3 × 10^4^) were seeded in 24-well plates before being transduced by spinfection with serial 1:4 dilutions of VLPs in the presence of 8 µg/ml polybrene (Merck). After 24 h, the media was replaced, with or without 15 µg/ml blasticidin. Four days post-transduction, cell viability was measured using CellTiter-Glo (Promega). Virus titers were calculated based on the dilution yielding < 30% transduction efficiency. Brunello library VLPs: MCF7 cells (3 × 10^6^) were seeded in 12-well plates before being transduced with serial 1:4 dilutions of VLPs in the presence of 8 µg/ml of polybrene. After 24 h, cells were re-seeded into duplicate 24-well plates with or without 1–2 µg/ml puromycin. Three days post-transduction, cell viability was measured using Cell-titer Glo. Virus titers were calculated based on the dilution yielding < 30% transduction efficiency.

### Pooled CRISPR-Cas9 screens

MCF7 cells were transduced with Lenti-Cas9‐2 A‐Blast VLPs at a multiplicity of infection (MOI) of 0.7 to minimize the potential immune response associated with Cas9 expression. After 24 h, cells were treated with 15 µg/ml blasticidin for two weeks to stabilize Cas9 expression, then maintained with 10 µg/ml blasticidin. MCF7-Cas9-expressing cells (1.3 × 10^8^ per replicate) were transduced with the Brunello CRISPR library at a MOI of 0.3 (500-cell coverage per gRNA). Three biological replicates were performed. Seven days post-selection, each replicate was split into two treatment groups: IVT *LacZ* or IVT *BRRIAR*, and transfected at 10 pM using Lipofectamine 3000. Three days post-transfection, genomic DNA was extracted from cells using the NucleoSpin Blood L midiprep kit (Macherey-Nagel) and integrated gRNA sequences were PCR amplified and barcoded using Q5 polymerase (NEB). PCR products were gel purified and sequenced by next-generation sequencing (20 × 10^6^ reads/replicate).

### CRISPR-Cas9 screen analysis

Guide RNA abundances were quantified from raw sequencing reads using MAGeCK v0.5.9.4 [71]. The significance of IVT *BRRIAR*-dependent counts was calculated using maximum-likelihood estimation (MLE) implemented in the MLE module, with 10 rounds of permutation (each round corresponding to 2 × gene number). MCF7 gene copy number was corrected using copy number variations (CNV) array data from the Broad Institute Cancer Cell Line Encyclopedia (CCLE). gRNA abundances were normalized by counts of non-targeting control gRNAs. Experimental treatment conditions for MLE modelling were supplied as binary design matrices including variables for baseline, IVT *LacZ* and IVT *BRRIAR* exposure.

### Apoptosis assays

T47D cells were transfected with 0.5 nM IVT *LacZ* or IVT *BRRIAR* for 48 h. For RNase L rescue experiments, T47D cells were transfected with 100 nM dsi-CON or dsi-*RNASEL* for 24 h, followed by IVT *BRRIAR* transfection for 48 h. Cells were then trypsinized, fixed and stained using the Alexa Fluor 488 Annexin V/Dead Cell Apoptosis Kit (Thermo Fisher Scientific) as per the manufacturer’s protocol. The percentage of apoptotic cells was quantified on a LSR Fortessa flow cytometer and FACSDiva software (BD Biosciences).

### Lipid nanoparticle (LNP) encapsulation of *BRRIAR*

The full-length *BRRIAR* DNA template was designed with a 5’ CleanCap AG promoter and 3’ poly(T) tract and synthesized by GeneArt (Thermo Fisher Scientific). IVT was performed using T7 RNA polymerase and N1-methyl-pseudouridine (Thermo Fisher Scientific) to generate *BRRIAR* RNA from the DNA template. The synthesized RNA was encapsulated into LNPs using the NanoAssemblr^®^ IgniteTM instrument (Precision NanoSystems) by the BASE Facility at the University of Queensland [29]. The LNP formulation used a lipid concentration of 10 mg/mL and a lipid molar ratio of 50 ionisable (SM-102)/10 DSPC/38.5 cholesterol/1.5 DMG-PEG2000. After encapsulation, LNP-*BRRIAR* was dialyzed into 1x Tris-buffered saline (TBS; pH 7.4). Particle size and charge were measured using a Zetasizer Ultra (Malvern Panalytical).

### Animal experiments

Female NSG mice (6–8 weeks old; NOD.Cg-Prkdc^*scid*^ll2rg^*tm1Wjl*^/SzJ/Ozarc [Ozgene, WA]) were implanted with slow-release β-estradiol pellets for 7 days prior to inoculated with 9.45 × 10^5^ luciferase-expressing MCF7 (MCF7-Luc) cells prepared in 50% growth factor reduced Matrigel (Corning) in PBS. Cells were injected subcutaneously into the right mammary fat pad. Tumor growth was monitored by caliper measurements and bioluminescent imaging using a Xenogen IVIS Spectrum (Caliper Life Sciences). After three weeks (when tumors were ~ 50 mm^3^), mice were randomized into two treatment groups of eleven mice each: LNP-control (empty LNPs) and LNP-*BRRIAR*. LNPs were administered at 0.5 mg/kg via intratumoral injection every 3 days for 12 days. Two mice from each group were euthanized 6 h after the first treatment and 48 h after the final treatment for tumor harvesting to assess *BRRIAR* and ISG expression. The remaining mice were monitored until tumors reached ~ 500 mm^3^. Mice survival analysis was performed using the Kaplan-Meier survival curve.

### Single cells RNA sequencing (scRNAseq)

ScRNAseq data were derived from a ER+/HER2 + breast tumor [Human Invasive Ductal Carcinoma, 3’v3.1, 150 × 150, Single Cell Gene Expression Dataset by Cell Ranger 6.0.0, 10x Genomics, (2021, March 31)], non-small cell lung cancer [NSCLC (F), v2, 150 × 150, 5’ Single Cell Immune Profiling Dataset by Cell Ranger 2.2.0, 10x Genomics (2018, August 1)], glioblastoma [Human Glioblastoma Multiforme, 3’v3.1, 150 × 150, Single Cell Gene Expression Dataset by Cell Ranger 6.0.0, 10x Genomics (2020, October 27)], ovarian tumor [Human Ovarian Tumor (FF), v2, 150 × 150, Single Cell Immune Profiling Dataset by Cell Ranger 7.0.0, 10x Genomics, (2022, May 14)] and pancreatic tumor [Pancreatic Tumor (FF), 5’v2, 150 × 150, Single Cell Immune Profiling Dataset by Cell Ranger 7.0.0, 10x Genomics (2022, May 14). Read counts were processed using the approach described in Wang et al. [72] to detect unannotated transcriptionally active regions. Data were analyzed and clustered using Seurat 4.3 [73]. Cell-types were assigned using scType [74], which maps clusters to known cell types. The ‘Unknown clusters’ were annotated using the top 250 marker genes per Seurat cluster with EnrichR, referencing the Human Gene Atlas (BioGPS) [75, 76] and CellMarker Augmented 2021. Correlation analysis of *BRRIAR* expression with *RIG-I* or *IRF7* was performed by calculating both Pearson correlation coefficient across cells with detectable *BRRIAR* (expression >0). Log-normalized expression values were obtained from the *Seurat* RNA assay (data slot). To ensure the analysis reflected true co-expression, cells with zero *IRF7* expression were excluded in a secondary analysis. Correlations were computed using the R cor() function. Scatter plots with regression lines, confidence intervals, and statistics were generated using the *ggpubr* package.

### Spatial transcriptomics

Spatial transcriptomic data were analyzed using two platforms: 10x Genomics Visium and Curio Seeker (Takara Bio USA). The data included an ER + breast tumor (Visium) [33], melanoma (Curio-Seeker, provided as a data release from Takara Bio), medulloblastoma [34] and colorectal tumor (Visium) [77]. The TAR-scRNA-seq pipeline was used to generate count matrices of annotated genes, including novel transcripts [72]. Reads mapping to the *BRRIAR* region were identified and their expression was spatially overlaid onto the tissue sections. *BRRIAR*-expressing cells in the Curio-Seeker dataset were identified by label transfer using Seurat [73], with an annotated scRNA-seq breast cancer reference dataset [33]. The spatial neighborhood composition of *BRRIAR*-positive cells in the breast cancer sample (Curio-Seeker dataset) was assessed using a permutation-based enrichment test. Thirteen *BRRIAR*-positive cells were identified among ~ 50,000 total cells. For each *BRRIAR*-positive cell, the 20 nearest neighbors were determined based on spatial coordinates using a k-d tree, and their cell-type identities were recorded. The average neighbor cell-type composition across all *BRRIAR*-positive cells was defined as the observed mean composition. To evaluate significance, we generated a null distribution by randomly sampling 13 cells from the full dataset 1,000 times and computing their mean neighbor compositions. Empirical *p*-values were calculated as the proportion of random means greater than or equal to the observed mean for each cell type. Cell types with *p* < 0.05 were considered significantly enriched in the *BRRIAR*-positive neighborhoods.

### Peripheral blood mononuclear cell (PBMC) preparation

PBMCs (5 × 10^5^ cells per donor) from Australian Red Cross Lifeblood were seeded into 96-well plates at 100 µl per well in phenol red-free RPMI 1640 with 20% FBS, 2x non-essential amino acids, 20 mM HEPES, 2 mM L-glutamine and 2% antibiotics. Cells were stimulated with 1 µg/ml HMCV pepmix before 100 µl cell culture supernatant from T47D transfected with 100 nM dsi-CON or dsi-*BRRIAR* plus 1 µg/ml 5’ppp-dsRNA, 0.5 nM IVT *LacZ* or 0.5 nM IVT *BRRIAR* were added. PBMC media and cells were harvested 48 h post-treatment and analyzed by flow cytometry.

### PBMC cytokine analysis

The LEGENDplex human type 1/2/3 interferon panel 5-plex (BioLegend) and the LEGENDplex custom human 13-plex panel (TNFα, TGFβ1, Perforin, IL-2, IL-7, IL-10, IL-6, IL-15, IL-12p70, Granzyme A, Granzyme B, CCL5, IFNγ) arrays were used for cytokine analysis as per the manufacturer’s protocol. Briefly, tissue culture supernatant and standards were mixed with antibody-immobilized beads in 96-well V-bottom plates and incubate for 2 h at 25 °C. Plates were centrifuged and washed before the beads were resuspended in detection antibody for 1 h. SA-PE reagent (Thermo Fisher Scientific) was then added to the wells and incubated for another 30 min. Plates were washed once and resuspended in washing buffer. Data were acquired using a LSR Fortessa flow cytometer and FACSDiva software (BD Biosciences). Analysis and calculations of cytokine concentrations were performed using the LEGENDplex Data Analysis Software Suite (BioLegend).

### Quantification and statistical analysis

Statistical calculations were performed using Prism software (GraphPad, v10). Student’s *t*-test, one- and two-way ANOVA analyses with Dunnett, Tukey, Sidak or Mantel-Cox tests were used to determine statistical significance as indicated in each figure legend. A *p* value of < 0.05 was considered significant in these analyses (*p < 0.05; **p < 0.01; ***p < 0.001, ****p < 0.0001).

## Supplementary Information

Supplementary Material 1.

Supplementary Material 2.

## Data Availability

The data generated in this study are publicly available in Gene Expression Omnibus (GEO) at GSE297682 and GSE305885.

## References

[1] Michailidou K, Lindstrom S, Dennis J, Beesley J, Hui S, Kar S, et al. Association analysis identifies 65 new breast cancer risk loci. Nature. 2017;551:92–4. 29059683 10.1038/nature24284 PMC5798588

[2] Fachal L, Aschard H, Beesley J, Barnes DR, Allen J, Kar S, et al. Fine-mapping of 150 breast cancer risk regions identifies 191 likely target genes. Nat Genet. 2020;52:56–73. 31911677 10.1038/s41588-019-0537-1 PMC6974400

[3] Beesley J, Sivakumaran H, Moradi Marjaneh M, Lima LG, Hillman KM, Kaufmann S, et al. Chromatin interactome mapping at 139 independent breast cancer risk signals. Genome Biol. 2020;21:8. 31910858 10.1186/s13059-019-1877-y PMC6947858

[4] Moradi Marjaneh M, Beesley J, O’Mara TA, Mukhopadhyay P, Koufariotis LT, Kazakoff S, et al. Non-coding RNAs underlie genetic predisposition to breast cancer. Genome Biol. 2020;21:7. 31910864 10.1186/s13059-019-1876-z PMC6947989

[5] Bitar M, Rivera IS, Almeida I, Shi W, Ferguson K, Beesley J, et al. Redefining normal breast cell populations using long noncoding RNAs. Nucleic Acids Res. 2023;51:6389–410. 37144467 10.1093/nar/gkad339 PMC10325898

[6] Statello L, Guo CJ, Chen LL, Huarte M. Gene regulation by long non-coding RNAs and its biological functions. Nat Rev Mol Cell Biol. 2021;22:96–118. 33353982 10.1038/s41580-020-00315-9 PMC7754182

[7] Wang L, Bitar M, Lu X, Jacquelin S, Nair S, Sivakumaran H, et al. CRISPR-Cas13d screens identify KILR, a breast cancer risk-associated LncRNA that regulates DNA replication and repair. Mol Cancer. 2024;23:101. 38745269 10.1186/s12943-024-02021-y PMC11094906

[8] McNab F, Mayer-Barber K, Sher A, Wack A, O’Garra A. Type I interferons in infectious disease. Nat Rev Immunol. 2015;15:87–103. 25614319 10.1038/nri3787 PMC7162685

[9] Zitvogel L, Galluzzi L, Kepp O, Smyth MJ, Kroemer G. Type I interferons in anticancer immunity. Nat Rev Immunol. 2015;15:405–14. 26027717 10.1038/nri3845

[10] Lazear HM, Schoggins JW, Diamond MS. Shared and distinct functions of type I and type III. Interferons Immun. 2019;50:907–23. 10.1016/j.immuni.2019.03.025 PMC6839410 30995506

[11] Kowalinski E, Lunardi T, McCarthy AA, Louber J, Brunel J, Grigorov B, et al. Structural basis for the activation of innate immune pattern-recognition receptor RIG-I by viral RNA. Cell. 2011;147:423–35. 22000019 10.1016/j.cell.2011.09.039

[12] Wang B, Wang Y, Pan T, Zhou L, Ran Y, Zou J, et al. Targeting a key disulfide linkage to regulate RIG-I condensation and cytosolic RNA-sensing. Nat Cell Biol. 2025;27:817–34. 40229436 10.1038/s41556-025-01646-5

[13] Elion DL, Jacobson ME, Hicks DJ, Rahman B, Sanchez V, Gonzales-Ericsson PI, et al. Therapeutically active RIG-I agonist induces Immunogenic tumor cell killing in breast cancers. Cancer Res. 2018;78:6183–95. 30224377 10.1158/0008-5472.CAN-18-0730

[14] Lin H, Jiang M, Liu L, Yang Z, Ma Z, Liu S, et al. The long noncoding RNA Lnczc3h7a promotes a TRIM25-mediated RIG-I antiviral innate immune response. Nat Immunol. 2019;20:812–23. 31036902 10.1038/s41590-019-0379-0

[15] Jiang M, Zhang S, Yang Z, Lin H, Zhu J, Liu L, et al. Self-Recognition of an inducible host LncRNA by RIG-I feedback restricts innate immune response. Cell. 2018;173:906–19. e13. 29706547 10.1016/j.cell.2018.03.064

[16] Lai C, Liu L, Liu Q, Wang K, Cheng S, Zhao L, et al. Long noncoding RNA AVAN promotes antiviral innate immunity by interacting with TRIM25 and enhancing the transcription of FOXO3a. Cell Death Differ. 2021;28:2900–15. 33990776 10.1038/s41418-021-00791-2 PMC8481484

[17] Fan J, Cheng M, Chi X, Liu X, Yang W. A human long Non-coding RNA LncATV promotes virus replication through restricting RIG-I-Mediated innate immunity. Front Immunol. 2019;10:1711. 31379885 10.3389/fimmu.2019.01711 PMC6658999

[18] Ke L, Yang DC, Wang Y, Ding Y, Gao G. AnnoLnc2: the one-stop portal to systematically annotate novel LncRNAs for human and mouse. Nucleic Acids Res. 2020;48:W230–8. 32406920 10.1093/nar/gkaa368 PMC7319567

[19] Zhao W, Zhang S, Zhu Y, Xi X, Bao P, Ma Z, et al. POSTAR3: an updated platform for exploring post-transcriptional regulation coordinated by RNA-binding proteins. Nucleic Acids Res. 2022;50:D287–94. 34403477 10.1093/nar/gkab702 PMC8728292

[20] Dumas L, Herviou P, Dassi E, Cammas A, Millevoi S. G-Quadruplexes in RNA biology: recent advances and future directions. Trends Biochem Sci. 2021;46:270–83. 33303320 10.1016/j.tibs.2020.11.001

[21] Sahayasheela VJ, Sugiyama H. RNA G-quadruplex in functional regulation of noncoding RNA: challenges and emerging opportunities. Cell Chem Biol. 2024;31:53–70. 37909035 10.1016/j.chembiol.2023.08.010

[22] Lee JH, Xiong F, Li W. Enhancer RNAs in cancer: regulation, mechanisms and therapeutic potential. RNA Biol. 2020;17:1550–9. 31916476 10.1080/15476286.2020.1712895 PMC7567500

[23] Corces MR, Granja JM, Shams S, Louie BH, Seoane JA, Zhou W et al. The chromatin accessibility landscape of primary human cancers. Science. 2018;362. 10.1126/science.aav1898 PMC6408149 30361341

[24] Tamura T, Yanai H, Savitsky D, Taniguchi T. The IRF family transcription factors in immunity and oncogenesis. Annu Rev Immunol. 2008;26:535–84. 18303999 10.1146/annurev.immunol.26.021607.090400

[25] Liu G, Park HS, Pyo HM, Liu Q, Zhou Y. Influenza A virus panhandle structure is directly involved in RIG-I activation and interferon induction. J Virol. 2015;89:6067–79. 25810557 10.1128/JVI.00232-15 PMC4442436

[26] Alexopoulou L, Holt AC, Medzhitov R, Flavell RA. Recognition of double-stranded RNA and activation of NF-kappaB by Toll-like receptor 3. Nature. 2001;413:732–8. 11607032 10.1038/35099560

[27] Osborne CK, Schiff R. Mechanisms of endocrine resistance in breast cancer. Annu Rev Med. 2011;62:233–47. 20887199 10.1146/annurev-med-070909-182917 PMC3656649

[28] Wienert B, Shin J, Zelin E, Pestal K, Corn JE. In vitro-transcribed guide RNAs trigger an innate immune response via the RIG-I pathway. PLoS Biol. 2018;16:e2005840. 30011268 10.1371/journal.pbio.2005840 PMC6049001

[29] Leighton LJ, Chaudhary N, Tompkins HT, Kulkarni A, Carrodus NL, Budzinska MA et al. The design, manufacture and LNP formulation of mRNA for research use. Nat Protoc. 2025. 10.1038/s41596-025-01174-4 40494942

[30] Boehmer DFR, Formisano S, de Oliveira Mann CC, Mueller SA, Kluge M, Metzger P et al. OAS1/RNase L executes RIG-I ligand-dependent tumor cell apoptosis. Sci Immunol 2021;6. 10.1126/sciimmunol.abe2550 34272227

[31] Chandrashekar DS, Karthikeyan SK, Korla PK, Patel H, Shovon AR, Athar M, et al. UALCAN: an update to the integrated cancer data analysis platform. Neoplasia. 2022;25:18–27. 35078134 10.1016/j.neo.2022.01.001 PMC8788199

[32] Banerjee S, Chakrabarti A, Jha BK, Weiss SR, Silverman RH. Cell-type-specific effects of RNase L on viral induction of beta interferon. mBio. 2014;5:e00856–14. 24570368 10.1128/mBio.00856-14 PMC3940032

[33] Wu SZ, Al-Eryani G, Roden DL, Junankar S, Harvey K, Andersson A, et al. A single-cell and spatially resolved atlas of human breast cancers. Nat Genet. 2021;53:1334–47. 34493872 10.1038/s41588-021-00911-1 PMC9044823

[34] Vo T, Balderson B, Jones K, Ni G, Crawford J, Millar A, et al. Spatial transcriptomic analysis of Sonic Hedgehog Medulloblastoma identifies that the loss of heterogeneity and promotion of differentiation underlies the response to CDK4/6 Inhibition. Genome Med. 2023;15:29. 37127652 10.1186/s13073-023-01185-4 PMC10150495

[35] Kawamata F, Patch AM, Nones K, Bond C, McKeone D, Pearson SA, et al. Copy number profiles of paired primary and metastatic colorectal cancers. Oncotarget. 2018;9:3394–405. 29423054 10.18632/oncotarget.23277 PMC5790471

[36] Stanton SE, Adams S, Disis ML. Variation in the incidence and magnitude of Tumor-Infiltrating lymphocytes in breast cancer subtypes: A systematic review. JAMA Oncol. 2016;2:1354–60. 27355489 10.1001/jamaoncol.2016.1061

[37] Sobral-Leite M, Van de Vijver K, Michaut M, van der Linden R, Hooijer GKJ, Horlings HM, et al. Assessment of PD-L1 expression across breast cancer molecular subtypes, in relation to mutation rate, BRCA1-like status, tumor-infiltrating immune cells and survival. Oncoimmunology. 2018;7:e1509820. 30524905 10.1080/2162402X.2018.1509820 PMC6279322

[38] Kiss Z, Mudryj M, Ghosh PM. Non-circadian aspects of BHLHE40 cellular function in cancer. Genes Cancer. 2020;11:1–19. 32577154 10.18632/genesandcancer.201 PMC7289903

[39] Cook ME, Jarjour NN, Lin CC, Edelson BT. Transcription factor Bhlhe40 in immunity and autoimmunity. Trends Immunol. 2020;41:1023–36. 33039338 10.1016/j.it.2020.09.002 PMC7606821

[40] Miao H, Wang L, Zhan H, Dai J, Chang Y, Wu F, et al. A long noncoding RNA distributed in both nucleus and cytoplasm operates in the PYCARD-regulated apoptosis by coordinating the epigenetic and translational regulation. PLoS Genet. 2019;15:e1008144. 31086376 10.1371/journal.pgen.1008144 PMC6534332

[41] DeVaux RS, Ropri AS, Grimm SL, Hall PA, Herrera EO, Chittur SV, et al. Long noncoding RNA BHLHE40-AS1 promotes early breast cancer progression through modulating IL-6/STAT3 signaling. J Cell Biochem. 2020;121:3465–78. 31907974 10.1002/jcb.29621 PMC7263938

[42] Barriocanal M, Prior C, Suarez B, Unfried JP, Razquin N, Hervas-Stubbs S, et al. Long noncoding RNA EGOT responds to stress signals to regulate cell inflammation and growth. J Immunol. 2021;206:1932–42. 33789981 10.4049/jimmunol.1900776

[43] Mousavi K, Zare H, Dell’orso S, Grontved L, Gutierrez-Cruz G, Derfoul A, et al. eRNAs promote transcription by Establishing chromatin accessibility at defined genomic loci. Mol Cell. 2013;51:606–17. 23993744 10.1016/j.molcel.2013.07.022 PMC3786356

[44] Ma S, Wang Z, Xiong Z, Ge Y, Xu MY, Zhang J, et al. Enhancer transcription profiling reveals an enhancer RNA-driven ferroptosis and new therapeutic opportunities in prostate cancer. Signal Transduct Target Ther. 2025;10:87. 40082405 10.1038/s41392-025-02170-6 PMC11906896

[45] Wang KC, Yang YW, Liu B, Sanyal A, Corces-Zimmerman R, Chen Y, et al. A long noncoding RNA maintains active chromatin to coordinate homeotic gene expression. Nature. 2011;472:120–4. 21423168 10.1038/nature09819 PMC3670758

[46] Gack MU, Shin YC, Joo CH, Urano T, Liang C, Sun L, et al. TRIM25 RING-finger E3 ubiquitin ligase is essential for RIG-I-mediated antiviral activity. Nature. 2007;446:916–20. 17392790 10.1038/nature05732

[47] Oshiumi H, Miyashita M, Inoue N, Okabe M, Matsumoto M, Seya T. The ubiquitin ligase riplet is essential for RIG-I-dependent innate immune responses to RNA virus infection. Cell Host Microbe. 2010;8:496–509. 21147464 10.1016/j.chom.2010.11.008

[48] Fox AH, Nakagawa S, Hirose T, Bond CS. Paraspeckles: where long noncoding RNA Meets phase separation. Trends Biochem Sci. 2018;43:124–35. 29289458 10.1016/j.tibs.2017.12.001

[49] Lee S, Kopp F, Chang TC, Sataluri A, Chen B, Sivakumar S, et al. Noncoding RNA NORAD regulates genomic stability by sequestering PUMILIO proteins. Cell. 2016;164:69–80. 26724866 10.1016/j.cell.2015.12.017 PMC4715682

[50] Jiang X, Muthusamy V, Fedorova O, Kong Y, Kim DJ, Bosenberg M, et al. Intratumoral delivery of RIG-I agonist SLR14 induces robust antitumor responses. J Exp Med. 2019;216:2854–68. 31601678 10.1084/jem.20190801 PMC6888973

[51] Moreno V, Calvo E, Middleton MR, Barlesi F, Gaudy-Marqueste C, Italiano A, et al. Treatment with a retinoic acid-inducible gene I (RIG-I) agonist as monotherapy and in combination with pembrolizumab in patients with advanced solid tumors: results from two phase 1 studies. Cancer Immunol Immunother. 2022;71:2985–98. 35596791 10.1007/s00262-022-03191-8 PMC10991664

[52] Peng B, Nguyen TM, Jayasinghe MK, Gao C, Pham TT, Vu LT, et al. Robust delivery of RIG-I agonists using extracellular vesicles for anti-cancer immunotherapy. J Extracell Vesicles. 2022;11:e12187. 35430766 10.1002/jev2.12187 PMC9013404

[53] Rady T, Erb S, Deddouche-Grass S, Morales R, Chaubet G, Cianferani S, et al. Targeted delivery of immune-stimulating bispecific RNA, inducing apoptosis and anti-tumor immunity in cancer cells. iScience. 2024;27:109068. 38380254 10.1016/j.isci.2024.109068 PMC10877685

[54] Toouli CD, Huschtscha LI, Neumann AA, Noble JR, Colgin LM, Hukku B, et al. Comparison of human mammary epithelial cells immortalized by Simian virus 40 T-Antigen or by the telomerase catalytic subunit. Oncogene. 2002;21:128–39. 11791183 10.1038/sj.onc.1205014

[55] El-Sahwi K, Bellone S, Cocco E, Cargnelutti M, Casagrande F, Bellone M, et al. In vitro activity of Pertuzumab in combination with trastuzumab in uterine serous papillary adenocarcinoma. Br J Cancer. 2010;102:134–43. 19920829 10.1038/sj.bjc.6605448 PMC2813756

[56] Netanely D, Avraham A, Ben-Baruch A, Evron E, Shamir R. Expression and methylation patterns partition luminal-A breast tumors into distinct prognostic subgroups. Breast Cancer Res. 2016;18:74. 27386846 10.1186/s13058-016-0724-2 PMC4936004

[57] Aran D, Sirota M, Butte AJ. Systematic pan-cancer analysis of tumour purity. Nat Commun. 2015;6:8971. 26634437 10.1038/ncomms9971 PMC4671203

[58] Thorsson V, Gibbs DL, Brown SD, Wolf D, Bortone DS, Ou Yang TH, et al. Immune Landsc Cancer Immun. 2018;48:812–30. e14. 10.1016/j.immuni.2018.03.023 PMC5982584 29628290

[59] Heath AP, Ferretti V, Agrawal S, An M, Angelakos JC, Arya R, et al. NCI Genomic Data Commons Nat Genet. 2021;53:257–62. 33619384 10.1038/s41588-021-00791-5

[60] Ghoussaini M, Edwards SL, Michailidou K, Nord S, Cowper-Sal Lari R, Desai K, et al. Evidence that breast cancer risk at the 2q35 locus is mediated through IGFBP5 regulation. Nat Commun. 2014;4:4999. 25248036 10.1038/ncomms5999 PMC4321900

[61] Martin M. Cutadapt removes adapter sequences from High-Throughput sequencing reads. EMBnet J. 2011;17:10–2.

[62] Langmead B, Salzberg SL. Fast gapped-read alignment with bowtie 2. Nat Methods. 2012;9:357–9. 22388286 10.1038/nmeth.1923 PMC3322381

[63] Zhang Y, Liu T, Meyer CA, Eeckhoute J, Johnson DS, Bernstein BE, et al. Model-based analysis of ChIP-Seq (MACS). Genome Biol. 2008;9:R137. 18798982 10.1186/gb-2008-9-9-r137 PMC2592715

[64] Ramirez F, Dundar F, Diehl S, Gruning BA, Manke T. DeepTools: a flexible platform for exploring deep-sequencing data. Nucleic Acids Res. 2014;42:W187–91. 24799436 10.1093/nar/gku365 PMC4086134

[65] Yu G, He QY. ReactomePA: an R/Bioconductor package for reactome pathway analysis and visualization. Mol Biosyst. 2016;12:477–9. 26661513 10.1039/c5mb00663e

[66] Bolger AM, Lohse M, Usadel B. Trimmomatic: a flexible trimmer for illumina sequence data. Bioinformatics. 2014;30:2114–20. 24695404 10.1093/bioinformatics/btu170 PMC4103590

[67] Dobin A, Davis CA, Schlesinger F, Drenkow J, Zaleski C, Jha S, et al. STAR: ultrafast universal RNA-seq aligner. Bioinformatics. 2013;29:15–21. 23104886 10.1093/bioinformatics/bts635 PMC3530905

[68] Li H, Handsaker B, Wysoker A, Fennell T, Ruan J, Homer N, et al. The sequence Alignment/Map format and samtools. Bioinformatics. 2009;25:2078–9. 19505943 10.1093/bioinformatics/btp352 PMC2723002

[69] Li B, Dewey CN. RSEM: accurate transcript quantification from RNA-Seq data with or without a reference genome. BMC Bioinformatics. 2011;12:323. 21816040 10.1186/1471-2105-12-323 PMC3163565

[70] Robinson MD, Smyth GK. Small-sample Estimation of negative binomial dispersion, with applications to SAGE data. Biostatistics. 2008;9:321–32. 17728317 10.1093/biostatistics/kxm030

[71] Li W, Xu H, Xiao T, Cong L, Love MI, Zhang F, et al. MAGeCK enables robust identification of essential genes from genome-scale CRISPR/Cas9 knockout screens. Genome Biol. 2014;15:554. 25476604 10.1186/s13059-014-0554-4 PMC4290824

[72] Wang MFZ, Mantri M, Chou SP, Scuderi GJ, McKellar DW, Butcher JT, et al. Uncovering transcriptional dark matter via gene annotation independent single-cell RNA sequencing analysis. Nat Commun. 2021;12:2158. 33846360 10.1038/s41467-021-22496-3 PMC8042062

[73] Hao Y, Hao S, Andersen-Nissen E, Mauck WM 3rd, Zheng S, Butler A, et al. Integrated analysis of multimodal single-cell data. Cell. 2021;184:3573–87. e29. 34062119 10.1016/j.cell.2021.04.048 PMC8238499

[74] Ianevski A, Giri AK, Aittokallio T. Fully-automated and ultra-fast cell-type identification using specific marker combinations from single-cell transcriptomic data. Nat Commun. 2022;13:1246. 35273156 10.1038/s41467-022-28803-w PMC8913782

[75] Su AI, Wiltshire T, Batalov S, Lapp H, Ching KA, Block D, et al. A gene atlas of the mouse and human protein-encoding transcriptomes. Proc Natl Acad Sci U S A. 2004;101:6062–7. 15075390 10.1073/pnas.0400782101 PMC395923

[76] Wu C, Orozco C, Boyer J, Leglise M, Goodale J, Batalov S, et al. BioGPS: an extensible and customizable portal for querying and organizing gene annotation resources. Genome Biol. 2009;10:R130. 19919682 10.1186/gb-2009-10-11-r130 PMC3091323

[77] Prakrithi P, Vo T, Vu H, Xiong A, Nguyen L, Newman A et al. Unraveling LncRNA diversity at a single cell resolution and in a Spatial context across different cancer types. bioRxiv 2024;2024.08.12.607523. 10.1038/s41592-026-03071-4 PMC13259954 42135486

