# Supplementary material for: BRRIAR lncRNA alters breast cancer risk by modulating interferon signaling in cis and in trans
Source: Mol Cancer. 2026 Jan 7;25:5. doi: 10.1186/s12943-025-02510-8 (PMC12777463; doi:10.1186/s12943-025-02510-8)
Supplement: Supplementary file 1 — Supplementary Material 1. [file 12943_2025_2510_MOESM1_ESM.docx]

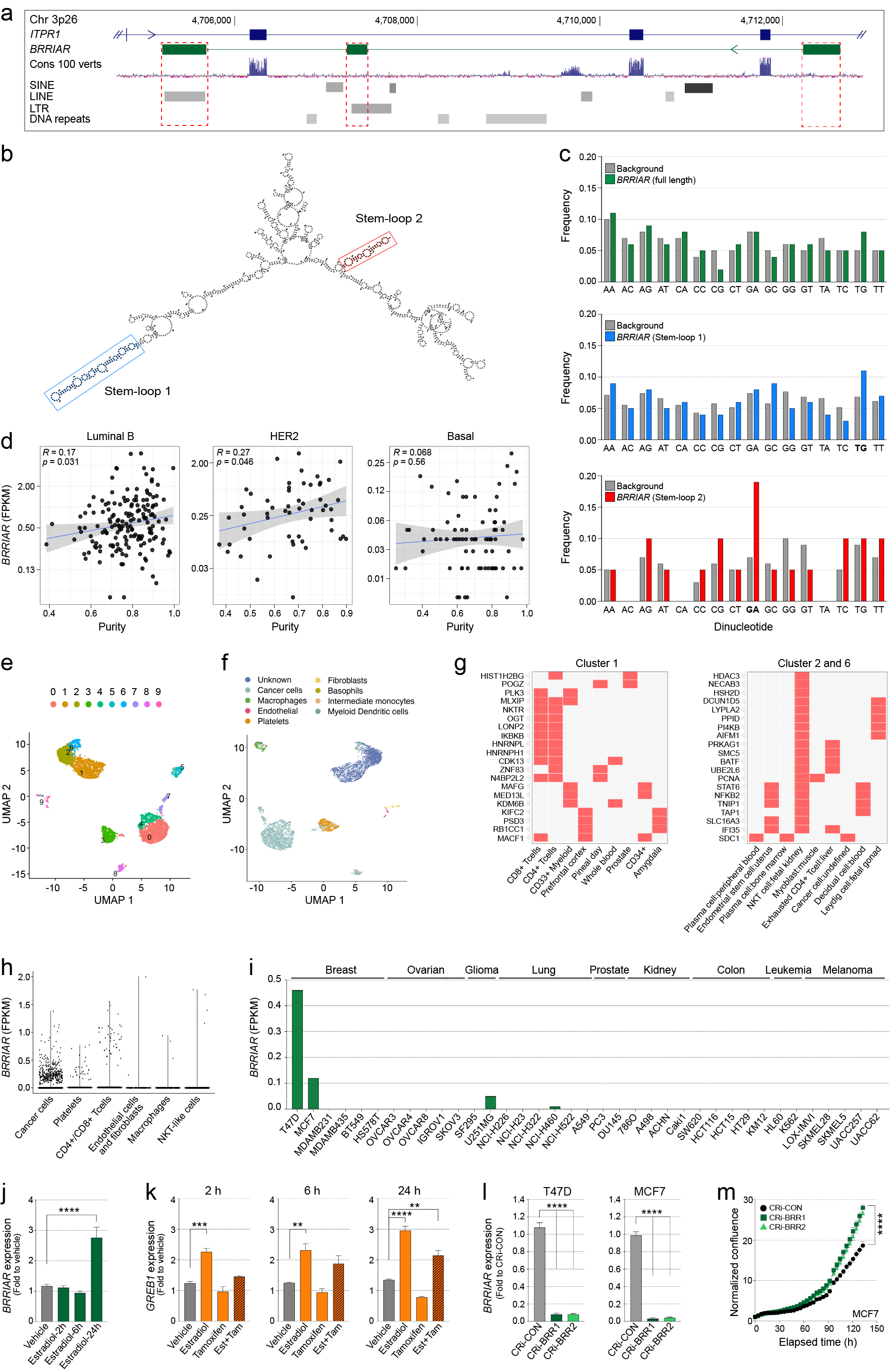


**Supplementary Figure 1.** **a** WashU genome browser (hg38) showing *ITPR1* (blue) and *BRRIAR* (green). The 100 vertebrates basewise conservation by PhyloP track is shown as a blue histogram. Transposable elements are marked as gray boxes; short/long interspersed nuclear elements (SINE/LINE), long terminal repeat elements (LTR) and DNA repeats. The dashed red outlines mark *BRRIAR* exons 1-3. **b** The predicted secondary structure of *BRRIAR* (generated using RNAfold). Red and blue outlines indicate two predicted long stem-loop structures. **c** Dinucleotide frequencies (calculated using PROG: [http://github.com/yinacobian/ frap/ blob/](http://github.com/yinacobian/%20frap/%20blob/)master/di_nt_freq.pl) for *BRRIAR* (top panel; green bars) and the predicted stem-loop structures (bottom panels; blue and red bars) compared with the expected frequencies from the local background (gray bars). **d** Scatter plots of *BRRIAR* FPKM (fragments per kilobase million) in luminal B, HER2 and basal breast tumors from TCGA plotted against estimated tumor purity. The correlation coefficient was calculated using Pearson correlation (*R*), and *p* values were determined using a two-sided *t*-test. **e** UMAP plot of Seurat clusters. **f** Cell-type assignment of the clusters using scType. **g** Inference of Seurat clusters 1, 2 and 6 using EnrichR of the top 250 cluster-specific markers. **h** Dot plot of *BRRIAR* FPKM across *BRRIAR*-positive cell types in one ER+/HER2+ invasive ductal carcinoma sample. **i** Normalized *BRRIAR* FPKM from AnnoLnc2 in the Cancer Cell Line Encyclopedia (CCLE) and ENCODE cancer cell lines. **j** qPCR for *BRRIAR* expression in T47D cells cultured in charcoal-stripped fetal bovine serum (CS-FBS) for 48 h, followed by treatment with estradiol (10 nM) for 2, 6 and 24 h. DMSO was the vehicle control. *GAPDH* was used as the qPCR internal control. Error bars, SEM (n = 3). *p* value was determined by one-way ANOVA with Dunnett’s test (****p < 0.0001). **k** qPCR for *GREB1* expression (positive control) in T47D cells cultured in CS-FBS for 48 h, followed by treatment with estradiol (10 nM), tamoxifen (1 μM) or estradiol + tamoxifen for 2, 6 and 24 h. DMSO was the vehicle control. *EIF2B1* was used as the qPCR internal control. Error bars, SEM (n = 3). *p* values were determined by one-way ANOVA with Dunnett’s test (**p < 0.01, ***p < 0.001, ****p < 0.0001). **l** qPCR for *BRRIAR* expression in T47D and MCF7 cells after CRISPRi-*BRRIAR*. The CRi-CON is a non-targeting control. *GAPDH* was used as the internal control. Error bars, SEM (n = 3). *p* values were determined by one-way ANOVA with Dunnett’s test (****p < 0.0001). **m** Cell confluence in MCF7 cells measured by IncuCyte after CRISPRi-*BRRIAR*. CRi-CON is a non-targeting control. Error bars, SEM (n = 8). *p* values were determined by one-way ANOVA with Dunnett’s test (****p < 0.0001).


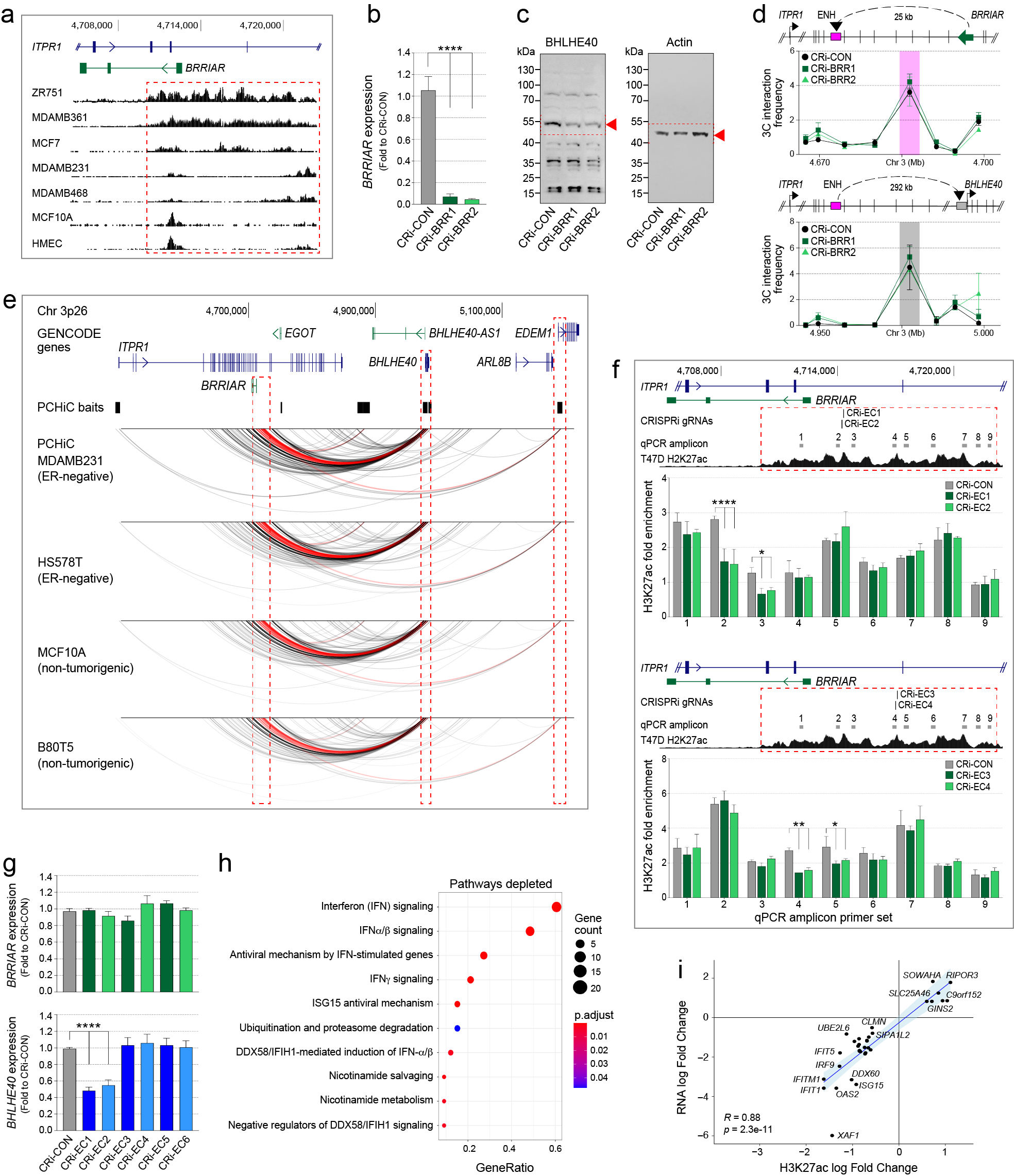


**Supplementary Figure 2. a** WashU genome browser (hg38) showing *ITPR1* (blue) and *BRRIAR* (green). H3K27ac tracks for ER+ (ZR751, MDAMB361, MCF7), ER- (MDAMB231, MDAMB468) and non-tumorigenic (MCF10A, HMEC) breast cell lines are shown as black histograms. The dashed red outline marks the ~11kb enhancer cluster. **b** qPCR for *BRRIAR* expression in T47D cells after CRISPRi-*BRRIAR*. The CRi-CON is a non-targeting control. *GAPDH* was used as the internal control. Error bars, SEM (n = 3). *p* values were determined by one-way ANOVA with Dunnett’s test (****p < 0.0001). **c** Uncropped Western blots for BHLHE40 and Actin in T47D cells after CRi-CON or CRi-*BRRIAR* treatment. **d** 3C interaction profiles between *BRRIAR* and a nearby enhancer (ENH; top) or between the ENH and *BHLHE40* promoter (bottom) in T47D cells after CRISPRi-*BRRIAR*. Error bars, SEM (n = 3). **e** WashU genome browser (hg38) showing annotated genes (blue) and noncoding RNAs (green). The promoter capture HiC (PCHiC) baits are marked as black boxes. PCHiC interactions are shown as arcs. The dashed red outlines and red arcs highlight chromatin looping between the enhancer cluster and *BHLHE40* and *EDEM1*. **f** H3K27ac ChIP-qPCR in T47D cells after CRi-EC1-2 (top graph) or CRi-EC3-4 (bottom graph). WashU genome browser (hg38) showing *ITPR1* (blue) and *BRRIAR* (green). The CRi gRNAs are marked as black vertical lines. qPCR amplicons are shown as gray boxes and numbered 1-9. The dashed red outline marks the ~11kb enhancer cluster. Bar graph of H3K27ac enrichment across the enhancer cluster. Data were normalized to an input signal and IgG control. Error bars, SD (n = 2). **g** qPCR for *BRRIAR* or *BHLHE40* expression in T47D cells after CRISPRi of two independent H3K27ac peaks (CRi-EC1-4) and the 3’ end (CRi-EC5-6) of the 11 kb enhancer cluster. The CRi-CON is a non-targeting control. *GAPDH* was used as the internal control. Error bars, SEM (n = 3). **h** Dot plot of GO analysis of H3K27ac ChIPseq data from T47D cells after CRISPRi-*BRRIAR*. **i** Scatter plot showing H3K27ac versus RNA log-fold changes. Each black dot represents a gene within 5 kb of an H3K27ac peak. A linear regression line with 95% confidence interval (blue with shaded area) is overlaid. The correlation coefficient was calculated using Pearson correlation (*R*), and *p* values were determined using a two-sided *t*-test.


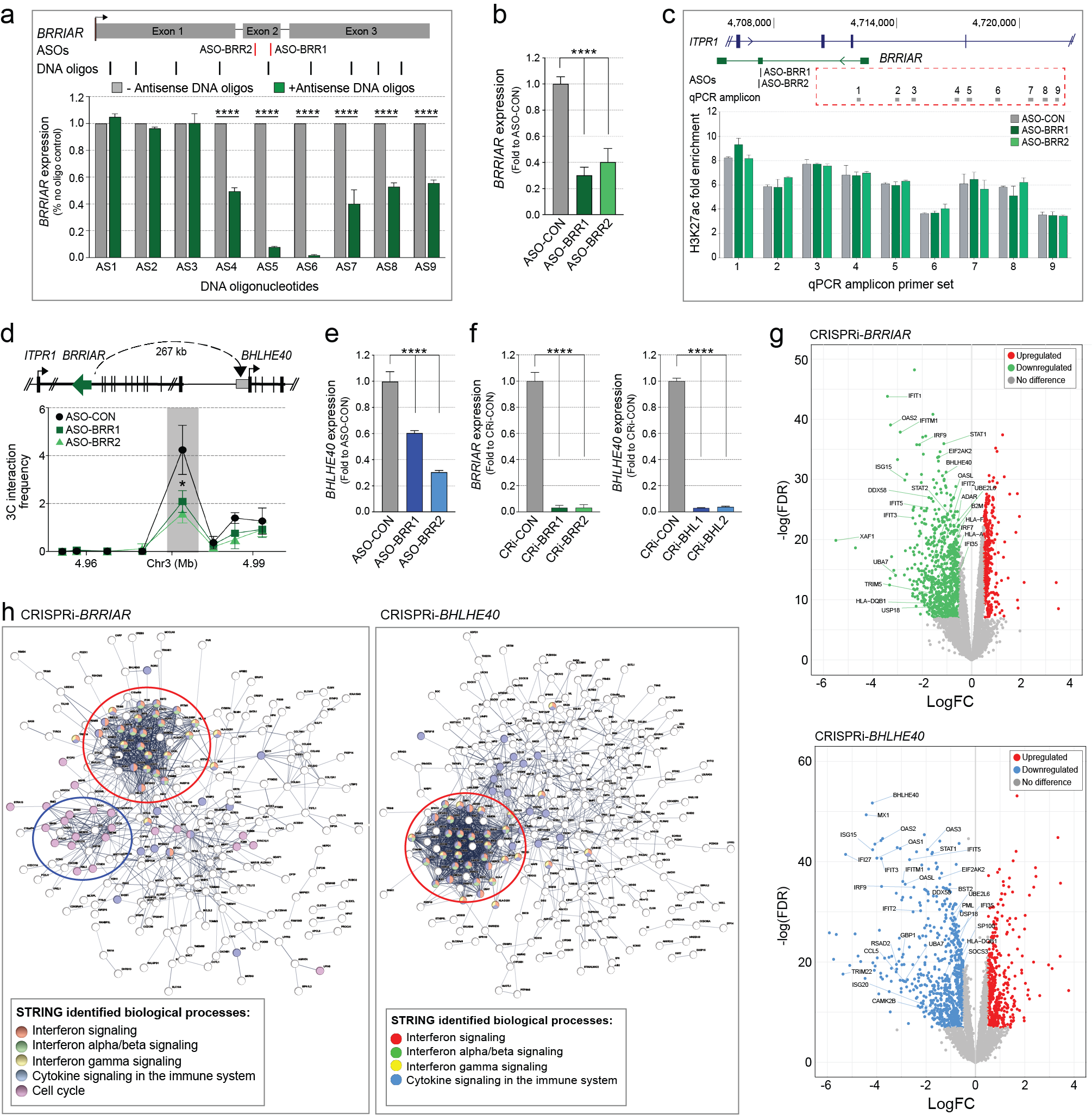


**Supplementary Figure 3*.*** **a** qPCR for *BRRIAR* expression after DNA oligonucleotides (AS1–9) targeting *BRRIAR* were incubated in T47D cellular extract and treated with RNase H. Schematic of the *BRRIAR* exons is shown above. Selected ASOs are marked as red vertical lines and DNA oligos AS1-9 as black vertical lines. Error bars, SD (n = 2). *p* values were determined by Student’s *t*-test (****p < 0.0001). **b** qPCR for *BRRIAR* expression in T47D cells after ASO-*BRRIAR*. The ASO-CON is a non-targeting control. *GAPDH* was used as the internal control. Error bars, SEM (n = 3). *p* values were determined by one-way ANOVA with Dunnett’s test (****p < 0.0001). **c** H3K27ac ChIP-qPCR in T47D cells after ASO-*BRRIAR*. Top panel: WashU genome browser (hg38) showing *ITPR1* (blue) and *BRRIAR* (green). The ASOs are marked as black vertical lines. qPCR primers are shown as gray boxes and numbered 1-9. The dashed red outline marks the ~11kb enhancer cluster. Bottom panel: H3K27ac enrichment across the 11 kb enhancer cluster. Data were normalized to an input signal and IgG control. Error bars, SD (n = 2). **d** 3C interaction profiles between *BRRIAR* and the *BHLHE40* promoter in T47Ds after ASO-*BRRIAR*. The ASO-CON is a non-targeting control. Error bars, SEM (n = 3). *p* value was determined by one-way ANOVA with Tukey test (*p < 0.05). **e** qPCR for *BHLHE40* expression in T47D cells after ASO-*BRRIAR*. *EIF2B1* was used as the internal control. Error bars, SEM (n = 3). *p* values were determined by one-way ANOVA with Dunnett’s test (****p < 0.0001). **f** qPCR for *BRRIAR* expression in T47D cells after CRISPRi-*BRRIAR* or *BHLHE40* expression after CRISPRi-*BHLHE40*. The CRi-CON is a non-targeting control. *GAPDH* was used as the internal control. Error bars, SEM (n = 3). *p* values were determined by one-way ANOVA with Dunnett’s test (****p < 0.0001). **g** Volcano plots showing differentially expressed genes (DEGs) in T47D cells after CRISPRi-*BRR2* or CRISPRi-*BHL2*. DEGs are shown as green, red or blue dots. **h** STRING interaction image for the top DEGs in T47D cells after CRISPRi-*BRRIAR* or CRISPRi-*BHLHE40*. The red circles highlight the downregulated DEGs, enriched for IFN signaling pathways. The blue circle indicates upregulated DEGs, enriched for cell cycle pathways.


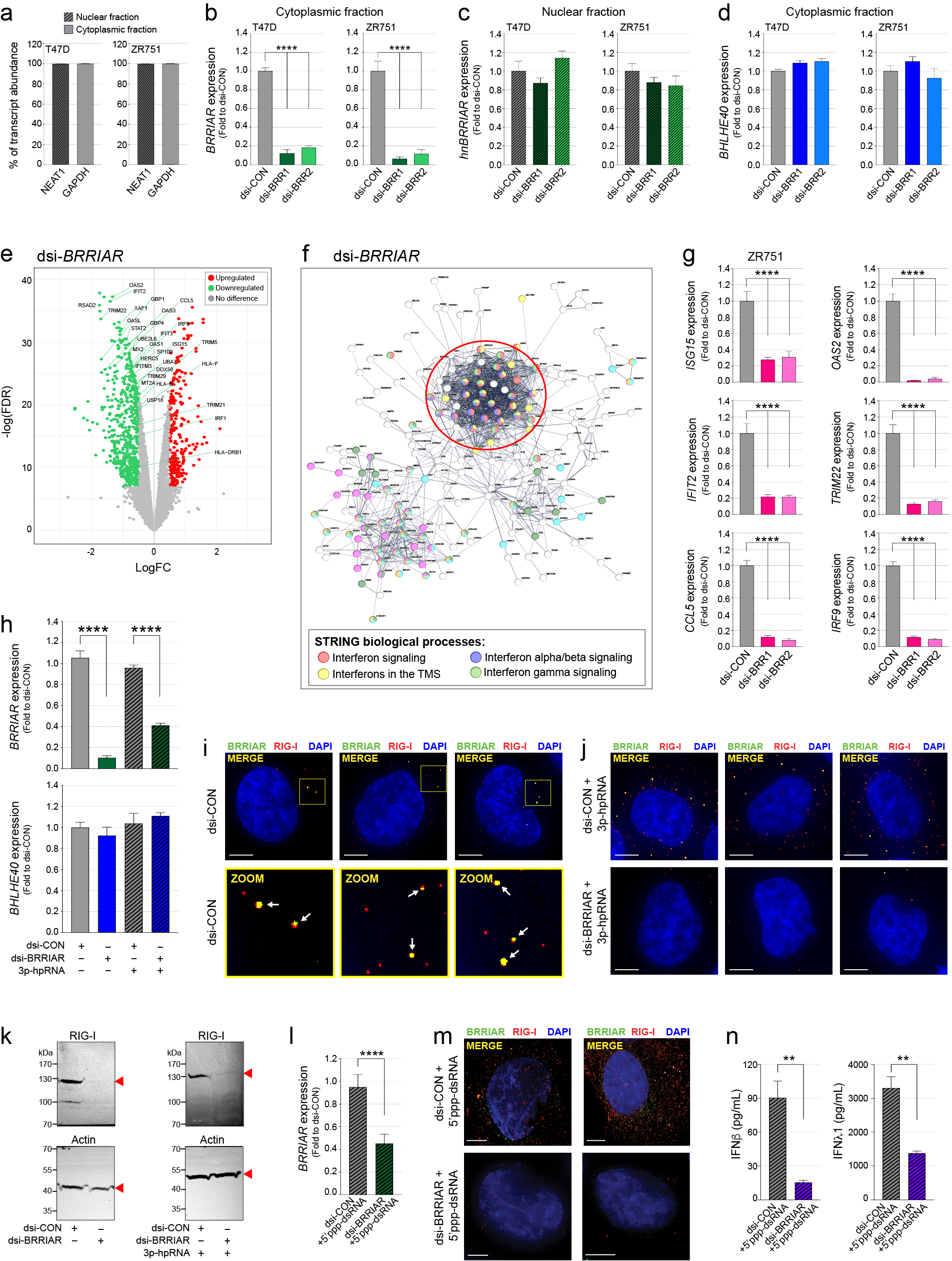


**Supplementary Figure 4.** **a** qPCR after nuclear/cytoplasmic fractionation of T47D and ZR751 cells detecting the distribution of *NEAT1* and *GAPDH* as nuclear and cytoplasmic markers, respectively. Error bars, SD (n = 2). **b-d** qPCR for *BRRIAR*, heterogenous nuclear (*hn*) *BRRIAR* and *BHLHE40* expression in T47D and ZR751 cells after dsi-*BRRIAR* treatment. The dsi-CON is a non-targeting control. *GAPDH* was used as the internal control. Error bars, SEM (n = 2). *p* values were determined by one-way ANOVA with Dunnett’s test (****p < 0.0001). **e** Volcano plot showing differentially expressed genes (DEGs) in T47D cells after dsi-*BRR2* treatment for 48 h. DEGs are shown as green or red dots. **f** STRING network interaction image for the top DEGs in T47D cells after dsi-*BRRIAR* treatment*.* The red outline indicates the downregulated DEGs after dsi-*BRRIAR*, enriched in IFN signaling pathways. **g** qPCR for ISGs in ZR751 cells after dsi-*BRRIAR* treatment. *GAPDH* was used as the internal control. Error bars, SEM (n = 3). *p* values were determined by one-way ANOVA with Dunnett’s test (****p < 0.0001). **h** qPCR for *BRRIAR* (top panel) and *BHLHE40* (bottom panel) expression in T47D cells after dsi-CON or dsi-*BRR1* treatment and exposure to 3p-hpRNA (0.5 μg/ml) for 6 h. *GAPDH* was used as the internal control. Error bars, SEM (n = 3). *p* values were determined by one-way ANOVA with Dunnett’s test (****p < 0.0001). **i** Additional microscopy images of *BRRIAR* (green) and RIG-I (red) in T47D cells after dsi-CON treatment. Nuclei were stained with DAPI (blue). Scale bars, 5 μm. White arrows highlight *BRRIAR*/RIG-I co-localization. **j** Additional microscopy images of *BRRIAR* (green) and RIG-I (red) in T47D cells after dsi-CON or dsi-*BRR1* treatment and exposure to 3p-hpRNA (0.5 μg/ml) for 6 h. Nuclei were stained with DAPI (blue). Scale bars, 5 μm. **k** Uncropped Western blots for RIG-I and Actin in T47D cells after dsi-CON or dsi-*BRR1* treatment and exposure to 3p-hpRNA. **l** qPCR for *BRRIAR* expression in T47D cells after dsi-CON or dsi-*BRR1* treatment and exposure to 5’ppp-dsRNA for 24 h. *GAPDH* was used as the internal control. Error bars, SEM (n = 3). *p* value was determined by Student’s *t-*test (****p < 0.0001). **m** Microscopy images of *BRRIAR* (green) and RIG-I (red) in T47D cells after dsi-CON or dsi-*BRR1* treatment and exposure to 5’ppp-dsRNA for 24 h. Nuclei were stained with DAPI (blue). Scale bars, 5 μm. **n** ELISA for IFNβ and IFNλ1 secreted from T47D cells after dsi-CON or dsi-*BRR1* treatment and exposure to 5’ppp-dsRNA for 24 h. Error bars, SEM (n = 3). *p* values were determined by Student’s *t-*test (**p < 0.01).

**
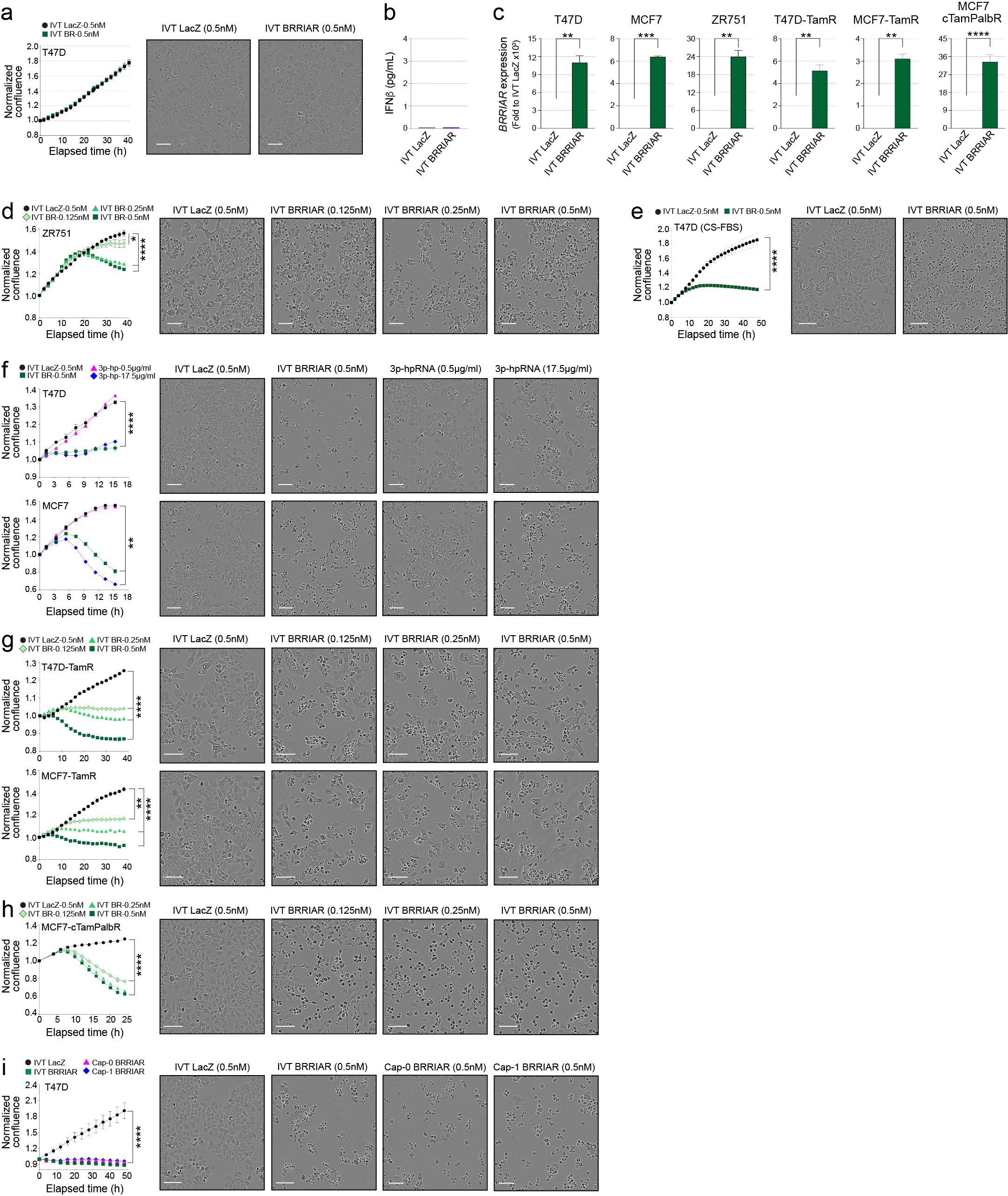
**

**Supplementary Figure 5. a** Left panel: cell confluence in T47D cells measured by IncuCyte following addition of IVT *LacZ* or IVT *BRRIAR* (0.5 nM) to the culture media. Error bars, SEM (n = 4). Right panels: representative IncuCyte images. Scale bar, 100 μm. **b** ELISA for IFNβ secreted from T47D cells after addition of IVT *LacZ* or IVT *BRRIAR* to the culture media for 40 h (n = 4). **c** qPCR for *BRRIAR* expression in breast cancer cell lines after transfection of IVT *LacZ* or IVT *BRRIAR* (0.5 nM) for 6 h. *GAPDH* was used as the internal control. Error bars, SD (n = 2). *p* values were determined by Student’s *t-*test (**p < 0.01, ***p < 0.001, ****p < 0.0001). **d** Left panel: cell confluence in ZR751 cells measured by IncuCyte after transfection of IVT *LacZ* (0.5 nM) or IVT *BRRIAR* (0.125 nM, 0.25 nM, 0.5 nM). Error bars, SEM (n = 4). *p* values were determined by one-way ANOVA with Dunnett’s test (*p < 0.05, ****p < 0.0001). Right panels: representative IncuCyte images. Scale bar, 100 μm. **e** Left panel: cell confluence in T47D cells cultured in CS-FBS for 48 h measured by IncuCyte after transfection of IVT *LacZ* (0.5 nM) or IVT *BRRIAR* (0.5 nM). Error bars, SEM (n = 4). *p* values were determined by one-way ANOVA with Dunnett’s test (****p < 0.0001). Right panels: representative IncuCyte images. Scale bar, 100 μm. **f** Left panels: cell confluence in T47D and MCF7 cells measured by IncuCyte after transfection with IVT *LacZ* (0.5 nM), IVT *BRRIAR* (0.5 nM) or 3p-hpRNA (0.5 μg/ml or 17.5 μg/ml). Error bars, SEM (n = 4). *p* values were determined by one-way ANOVA with Dunnett’s test (**p < 0.01, ****p < 0.0001). Right panels: representative IncuCyte images. Scale bar, 100 μm. **g** Cell confluence in T47D-TamR and MCF7-TamR cells measured by IncuCyte after transfection of IVT *LacZ* (0.5 nM) or IVT *BRRIAR* (0.125 nM, 0.25 nM, 0.5 nM). Error bars, SEM (n = 4). *p* values were determined by one-way ANOVA with Dunnett’s test (**p < 0.01, ****p < 0.0001). Right panels: representative IncuCyte images. Scale bar, 100 μm. **h** Cell confluence in MCF7-cTamPalbR cells measured by IncuCyte after transfection of IVT *LacZ* (0.5 nM) or IVT *BRRIAR* (0.125 nM, 0.25 nM, 0.5 nM). Error bars, SEM (n = 4). *p* values were determined by one-way ANOVA with Dunnett’s test (****p < 0.0001). Right panels: representative IncuCyte images. Scale bar, 100 μm. **i** Left panel: cell confluence in T47D cells measured by IncuCyte after transfection of IVT *LacZ*, IVT *BRRIAR* or Cap-0/Cap-1 *BRRIAR* (all 0.5 nM). Error bars, SEM (n = 4). *p* values were determined by one-way ANOVA with Dunnett’s test (****p < 0.0001). Right panels: representative IncuCyte images. Scale bar, 100 μm.


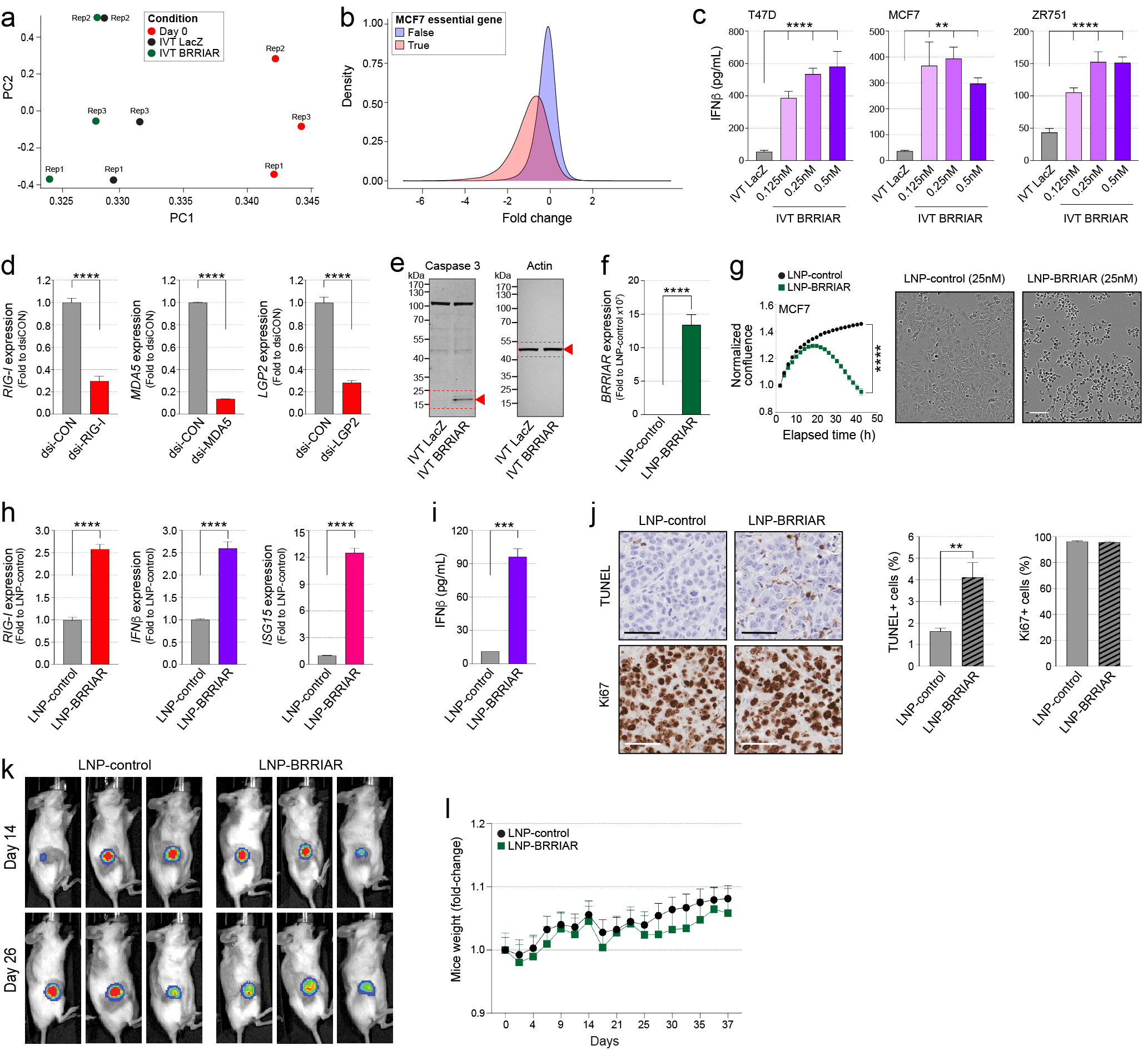


**Supplementary Figure 6. a** First and second principal components (PC) for normalized counts, with sample treatments colored and replicates (Rep1-3) labelled. **b** Distributions of log mean counts for guide RNAs targeting essential genes in MCF7 cells (orange) compared with non-essential genes (blue). **c** ELISA for IFNβ secreted from ER+ breast cancer cell lines after transfection of IVT *LacZ* (0.5 nM) or IVT *BRRIAR* (0.125 nM, 0.25 nM, 0.5 nM). Error bars, SD (n = 2). *p* values were determined by one-way ANOVA with Dunnett’s test (**p < 0.01, ****p < 0.0001). **d** qPCR for *RIG-I*, *MDA5* and *LGP2* expression in T47D cells after co-transfection with indicated dsiRNAs and IVT *BRRIAR* (0.5 nM). *GAPDH* was used as the internal control. Error bars, SEM (n = 4). *p* values were determined by Student’s *t-*test (****p < 0.0001). **e** Uncropped Western blots for caspase 3 and Actin (loading control) in T47D cells after transfection of IVT *LacZ* or IVT *BRRIAR* (0.2 nM) for 24 h. **f** qPCR for *BRRIAR* expression in MCF7 cells after the addition of LNP-control or LNP-*BRRIAR* (25 nM) for 6 h. *GAPDH* was used as the internal control. Error bars, SEM (n = 3). *p* value was determined by Student’s *t-*test (****p < 0.0001). **g** Left panel: cell confluence in MCF7 cells measured by IncuCyte after addition of LNP-control or LNP-*BRRIAR* (25 nM). Error bars, SEM (n = 4). *p* values were determined by one-way ANOVA with Dunnett’s test (****p < 0.0001). Right panels: representative IncuCyte images. Scale bar, 100 μm. **h** qPCR for ISG expression in MCF7 cells after addition of LNP-control or LNP-*BRRIAR* (25 nM) for 6 h. *GAPDH* was used as the internal control. Error bars, SEM (n = 3). *p* value was determined by Student’s *t-*test (****p < 0.0001). **i** ELISA for IFNβ secreted from MCF7 cells after addition of LNP-control or LNP-*BRRIAR* (25 nM; n = 2). *p* value was determined by Student’s *t-*test (***p < 0.001). **j** Left panels: representative images of IHC analysis showing TUNEL assay or Ki67 staining in MCF7 tumor sections on day 26 after I.T. injections of LNP-control or LNP-*BRRIAR* (0.5 mg/kg). Scale bar, 60 μm. Right panel: Percentage of TUNEL- or Ki67-positive cells derived from five random fields per sample (n = 2 mice per group). *p* value was determined by Student’s *t-*test (**p < 0.01). **k** Bioluminescence intensities of MCF7 tumors in mice imaged before (day 14) and after (day 26) intratumoral injections of LNP-control or LNP-*BRRIAR* (0.5 mg/kg). **l** Body weight of mice in both groups.

**
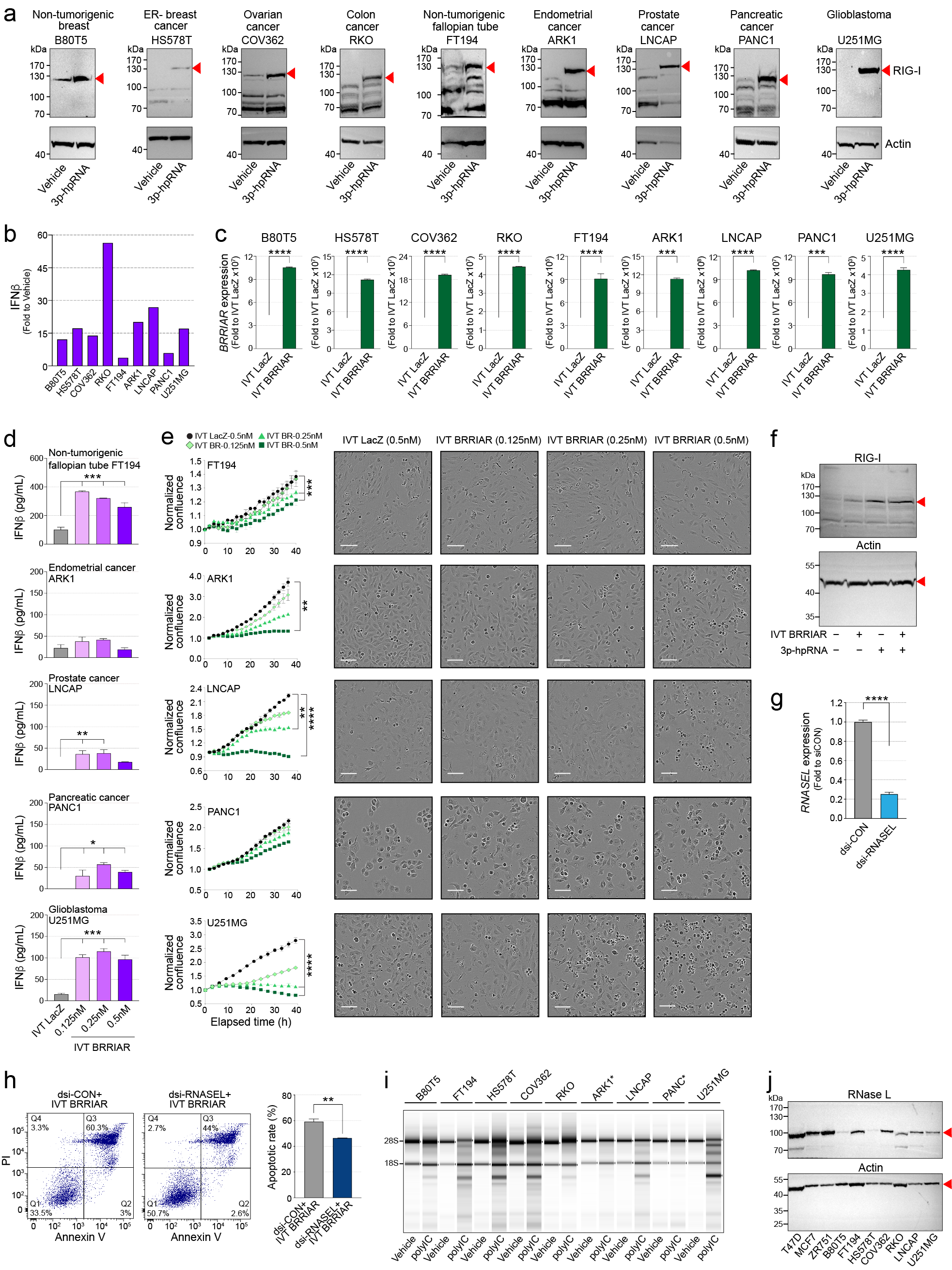
**

**Supplementary Figure 7. a** Western blots for RIG-I in a panel of cell lines after transfection of vehicle or 3p-hpRNA (0.5 μg/ml) for 24 h. Actin was used as the loading control. **b** ELISA for IFNβ secreted from cell lines after transfection of vehicle or 3p-hpRNA (0.5 μg/ml) for 24 h (n = 2). **c** qPCR for *BRRIAR* expression in cell lines after transfection of IVT *LacZ* or IVT *BRRIAR* (0.5 nM) for 6 h. *GAPDH* was used as the internal control. Error bars, SD (n = 2). *p* values were determined by Student’s *t-*test (***p < 0.001, ****p < 0.0001). **d** ELISA for IFNβ secreted from cell lines after transfection of IVT *LacZ* (0.5 nM) or IVT *BRRIAR* (0.125 nM, 0.25 nM, 0.5 nM). Error bars, SEM (n = 4). *p* values were determined by one-way ANOVA with Dunnett’s test (*p < 0.01, **p < 0.01, ***p < 0.001). **e** Left panels: cell confluence in cell lines measured by IncuCyte after transfection of IVT *LacZ* (0.5 nM) or IVT *BRRIAR* (0.125 nM, 0.25 nM, 0.5 nM). Error bars, SEM (n = 4). *p* values were determined by one-way ANOVA with Dunnett’s test (**p < 0.01, ***p < 0.001, ****p < 0.0001). Right panels: representative IncuCyte images. Scale bar, 100 μm. **f** Uncropped Western blots for RIG-I and Actin in B80T5 cells after transfection of IVT *BRRIAR* (0.5 nM) and/or 3p-hpRNA (0.5 μg/ml) for 6 h. **g** qPCR for *RNASEL* expression in T47D cells after co-transfection with dsi-CON or pooled dsi-*RNASEL* (dsi-*RNASEL1-3*) and IVT *BRRIAR* (0.5 nM) for 40 h. The dsi-CON is a non-targeting control. *EIF2B1* was used as the internal control. Error bars, SEM (n = 3). *p* value was determined by Student’s *t-*test (****p < 0.0001). **h** Left panels: apoptosis analysis of T47D cells after co-transfection with indicated dsiRNAs and IVT *BRRIAR* (0.5 nM) for 40 h by double staining with Annexin V (AV) and propidium iodide (PI). The quadrants (Q) were defined as Q1 = live (AV-/PI-negative), Q2 = early stage of apoptosis (AV-positive/PI-negative), Q3 = late stage of apoptosis (AV-/PI-positive) and Q4 = necrosis (AV-negative/PI-positive). Right panel: percentage of cells in early and late-stage apoptosis in each group (Q2 + Q3). Error bars, SEM (n = 3). *p* value was determined by Student’s *t-*test (**p < 0.01). **i** TapeStation rRNA integrity analysis of total RNA isolated from a panel of cell lines after transfection of polyIC (100 nM) for 24-40 h. *Cell lines with no detectable RNase L activity under the specified conditions. **j** Uncropped Western blots for RNase L and Actin in a panel of cancer cell lines.


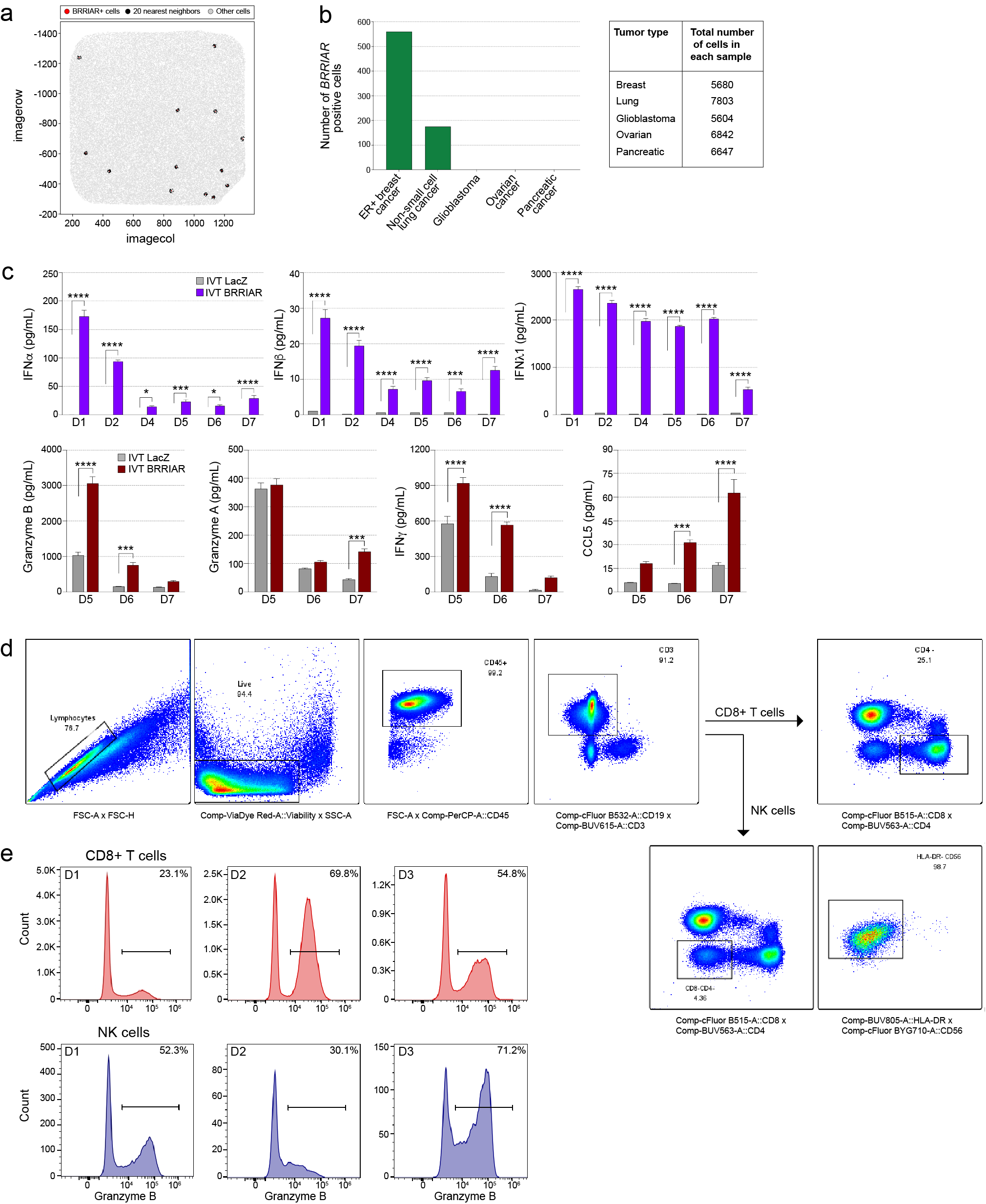


**Supplementary Figure 8. a** Spatial distribution of *BRRIAR*-positive cells (red dots), their 20 nearest neighbors (black dots) and *BRRIAR*-negative cells (gray dots) in an ER+ breast tumor sample. **b** Left panel: Number of *BRRIAR*-positive cells identified from single-cell RNAseq data across tumor types (10x Genomics). Right panel: Total number of cells in each tumor sample. **c** Bead-based profiling of cytokines secreted from HCMV-activated PBMCs (donors; D1-7) after 48 h exposure to media harvested from T47D transfected with IVT *LacZ* or IVT *BRRIAR* (0.5 nM) for 24 h. Error bars, SEM (n = 4). *p* values were determined by two-way ANOVA with Sidak’s test (*p < 0.05, ***p < 0.001, ****p < 0.0001). **d** Gating strategy to identify CD8+ T and natural killer (NK) cells from PBMCs. **e** Flow cytometry profiles of Granzyme B expression on CD8+ T-cells and NK cells from HCMV-activated PMBCs (D1-3) after 48 h exposure to media harvested from T47D cells transfected with dsi-CON. The histogram profiles highlight the cellular source of cytokines but the geometric mean intensity (GMI) or cellular percentage are not shown as cells were harvested after cytokines were secreted.
